# Stigmasterol attenuates atherosclerosis by inhibiting inflammatory signaling and foam cell formation

**DOI:** 10.1002/imo2.70056

**Published:** 2025-10-01

**Authors:** Baiyi Lu, Fan Xiao, Qinjun Zhang, Yao Xie, Mengmeng Wang, Jesus Simal‐Gandara, Yan Liu, Thomas Efferth, Weisu Huang, Jianfu Shen, Jianbo Xiao

**Affiliations:** ^1^ College of Biosystems Engineering and Food Science, National‐Local Joint Engineering Laboratory of Intelligent Food Technology and Equipment, Key Laboratory for Agro‐Products Nutritional Evaluation of Ministry of Agriculture and Rural Affairs, Key Laboratory of Agro‐Products Postharvest Handling of Ministry of Agriculture and Rural Affairs Zhejiang Key Laboratory for Agro‐Food Processing, Zhejiang International Scientific and Technological Cooperation Base of Health Food Manufacturing and Quality Control, Zhejiang University Hangzhou China; ^2^ ZJU‐Hangzhou Global Scientific and Innovation Center Zhejiang University Hangzhou China; ^3^ Department of Cardiology, The Second Affiliated Hospital Zhejiang University School of Medicine, Cardiovascular Key Lab of Zhejiang Province Hangzhou China; ^4^ Department of Analytical Chemistry and Food Science, Faculty of Food Science and Technology University of Vigo‐Ourense Campus Ourense Spain; ^5^ Department of Pharmaceutical Biology, Institute of Pharmacy and Biomedical Sciences Johannes Gutenberg University Staudinger Weg Mainz Germany; ^6^ Department of Applied Technology Zhejiang Economic & Trade Polytechnic Hangzhou China

**Keywords:** atherosclerosis, foam cell, inflammation, phenotype transformation, phytosterol, single‐cell RNA sequencing

## Abstract

Foam cells derived from macrophages and smooth muscle cells (SMCs) play a pivotal role in the progression of atherosclerosis. While phytosterols (PS) have demonstrated cholesterol‐lowering and anti‐inflammatory properties, their impact on foam cells remains elusive. Here, we investigated the effects of PS on foam cell formation, inflammatory responses, and lipid metabolism using both single‐cell RNA sequencing (scRNA‐seq) and functional assays. scRNA‐seq of aortic tissue from *ApoE* ^−/−^ mice revealed that PS supplementation reduced proinflammatory macrophages and SMC‐derived intermediate cells. Among the PS components, stigmasterol most effectively attenuated foam cell formation by suppressing oxidized low‐density lipoprotein (ox‐LDL) uptake and enhancing cholesterol efflux. Mechanistically, stigmasterol inhibited the inflammatory CD86^+^ macrophage polarization by activating the Adenosine Monophosphate‐Activated Protein Kinase (AMPK) pathway and inhibiting the NF‐κB/NLRP3 signaling axis. In SMCs, stigmasterol upregulated ABCA1 and ABCG1 expression, reduced lipid accumulation, and suppressed CD68 expression, thereby limiting trans‐differentiation into macrophage‐like foam cells. In vivo, stigmasterol reduced plaque burden, promoted anti‐inflammatory macrophage polarization, and inhibited SMC‐to‐macrophage transition in *ApoE* ^−/−^ mice. Collectively, these findings uncover a previously underexplored role of stigmasterol in modulating foam cell diversity and inflammation, providing mechanistic insight into the vascular protective effects of dietary PS.

## Highlights

- Single‐cell RNA sequencing (scRNA‐seq) revealed that dietary phytosterol supplementation reduces the proportion of inflammatory macrophages and smooth muscle cell (SMC)‐derived intermediate cells in the aortas of *ApoE* ^−/−^ mice.
- Stigmasterol promotes cholesterol efflux and suppresses the uptake of oxidized low‐density lipoprotein (ox‐LDL) in foam cells.
- Stigmasterol inhibits the polarization inflammatory of CD86^+^ macrophages through modulation of the AMPK/NF‐κB/NLRP3 signaling axis, and downregulates CD68 expression in SMCs, thereby contributing to the attenuation of vascular inflammation.

## Graphical abstract

Stigmasterol mitigates atherosclerotic plaque formation by modulating foam cell phenotypic alterations and cholesterol metabolism. Stigmasterol inhibits inflammatory CD86^+^ macrophage polarization via the NF‐κB/NLRP3 pathway and downregulates CD68 expression in smooth muscle cells (SMCs), contributing to reduced vascular inflammation. It also enhances cholesterol efflux and suppresses oxidized low‐density lipoprotein (ox‐LDL) uptake in foam cells. These findings offer potential therapeutic strategies for atherosclerosis prevention and treatment.

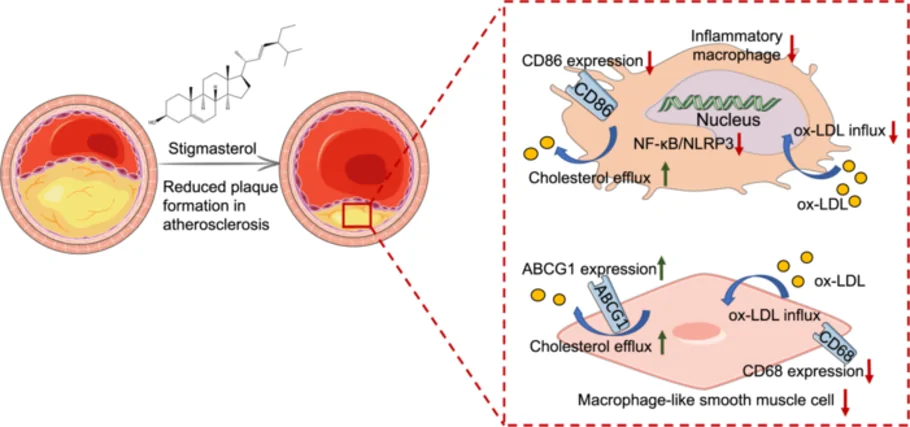

## 1 INTRODUCTION

Atherosclerosis (AS) is a chronic inflammatory disease driven by lipid accumulation and characterized by complex pathogenesis [1]. Foam cells, primarily derived from macrophages and smooth muscle cells (SMCs), play a crucial role in plaque formation and destabilization [2]. Foam cell homeostasis is tightly regulated by the balance between lipid uptake and cholesterol efflux. Uncontrolled uptake of oxidised low‐density lipoprotein (ox‐LDL) and impaired reverse cholesterol transport are key features, mediated by proteins involved in lipid metabolism. These include scavenger receptors such as SRA, and CD36, which promote lipid uptake, and cholesterol transporters like ABCA1 and ABCG1, which facilitate cholesterol efflux [3, 4].

The polarization of macrophages and SMCs is controlled by complex interactions among various factors [5, 6], affecting foam cell formation by modulating local inflammation and lipid accumulation [7]. Although M1 and M2 macrophages have been widely studied, their precise roles in plaque progression remain debated. Johnson et al reported that both M1 and M2 macrophages are involved in plaque formation and may aggravate AS [8]. However, the M1/M2 classification is largely derived from simplified in vitro systems, and macrophage phenotypes in vivo are more diverse and context‐dependent [9]. Recent studies have identified multiple macrophage subsets within atherosclerotic plaques, including *Trem2* ^*hi*^, resident‐like, and inflammatory macrophages, which emerge through reprogramming driven by lipid overload and inflammation [10]. SMCs also exhibit remarkable phenotypic plasticity. In response to vascular injury or pathological stimuli, they can transition from contractile to synthetic phenotype and further differentiate into osteoblasts, chondrocytes, adipocytes, and macrophage‐like foam cell phenotypes. These cells often retain SMC markers such as α‐SMA/ACTA2, despite acquiring new functional roles [11].

Phytosterols (PS), naturally occurring tetracyclic triterpenoids abundant in vegetable oils and grains [12], have been widely reported to exert anti‐inflammatory and cholesterol‐lowering effects [13, 14, 15]. However, their direct impact on foam cell formation and vascular health remains incompletely understood [16]. While previous studies have demonstrated PS benefit in lipid metabolism, their influence on foam cell heterogeneity at the single‐cell level has not been explored.

Single‐cell RNA sequencing (scRNA‐seq) technology provides unprecedented resolution to investigate cellular heterogeneity, enabling the profiling of individual cells isolated from complex tissues [17]. In this study, we utilized aorta cells from *ApoE* ^−/−^ mice treated with a 0.2 g/kg phytosterol mixture (β‐sitosterol, campesterol, and stigmasterol), following our laboratory's previously established protocol [18]. We first characterized the phenotypic changes of macrophages and SMCs in response to phytosterol intervention. Subsequently, we identified key molecular targets and signaling pathways modulated by PS, with a particular focus on stigmasterol, the principal component of the mixture. Finally, we validated the effects of stigmasterol on foam cell formation, lipid metabolism, and inflammation through a series of in vitro and in vivo experiments. Collectively, our findings offer mechanistic insights into the role of PS in regulating foam cell heterogeneity and identify stigmasterol as a promising therapeutic agent for AS.

## 2 RESULTS

### Identification of macrophage heterogeneity induced by PS

Building upon our previous research [18], we performed scRNA‐seq analysis of aortic tissue from *ApoE* ^−/−^ mice to investigate changes in cellular composition and gene expression following dietary supplementation with PS or their oxidation products, phytosterols oxidation products (POPs), and cholesterol oxidation products (COPs). Both PS and cholesterol are prone to oxidation, forming structurally distinct POPs and COPs, particularly in the B ring, which may critically alter their biological effects (Supporting Information S1: Figure S1A). To comprehensively assess the effects of PS and their oxidation products, we compared a PS mixture (β‐sitosterol, campesterol, and stigmasterol) with POPs and COPs in terms of their effects on atherosclerotic lesion formation and disease progression (Supporting Information S1: Table S1 and Supporting Information S1: Figure S1B). Compared with the control, COPs, and POPs groups, mice fed a PS‐enriched diet exhibited significantly reduced atherosclerotic lesion area (Supporting Information S1: Figure S1C–E) and marked decrease in total cholesterol (TC) and low‐density lipoprotein cholesterol (LDL‐C) levels (Supporting Information S1: Figure S1F) without affecting mice body weight (Supporting Information S1: Figure S2A). Furthermore, PS treatment reduced plasma levels of pro‐inflammatory cytokines TNF‐α and IL‐6, while increasing the anti‐inflammatory cytokine IL‐10 (Supporting Information S1: Figure S2B), suggesting a protective role for PS against AS.

For scRNA‐seq analysis, aortic tissues from 5 mice per group were collected, enzymatically dissociated, and subjected to single‐cell sorting after removal of perivascular adipose tissue. Live nucleated cells (Hoechst+/APC/Cy7−) were isolated for sequencing (Supporting Information S1: Figure S3). Following quality control, 2996 cells from the control group, 3034 from the PS group, 4936 from the COPs group, and 3914 from the POPs group were retained for downstream analysis. Unsupervised clustering using Seurat identified 20 distinct cell clusters, visualized using t‐distributed stochastic neighbor embedding (t‐SNE) (Supporting Information S1: Figures S4 and S5). Cell type annotation based on canonical marker genes revealed that clusters 4, 12, and 19 represented macrophages (expressing *C1qb* and *C1qa*), while clusters 10 and 14 were classified as SMCs expressing *Acta2*, *Myh11*, and *Tagln* (Supporting Information S1: Figure S4D).

**Figure 1:**
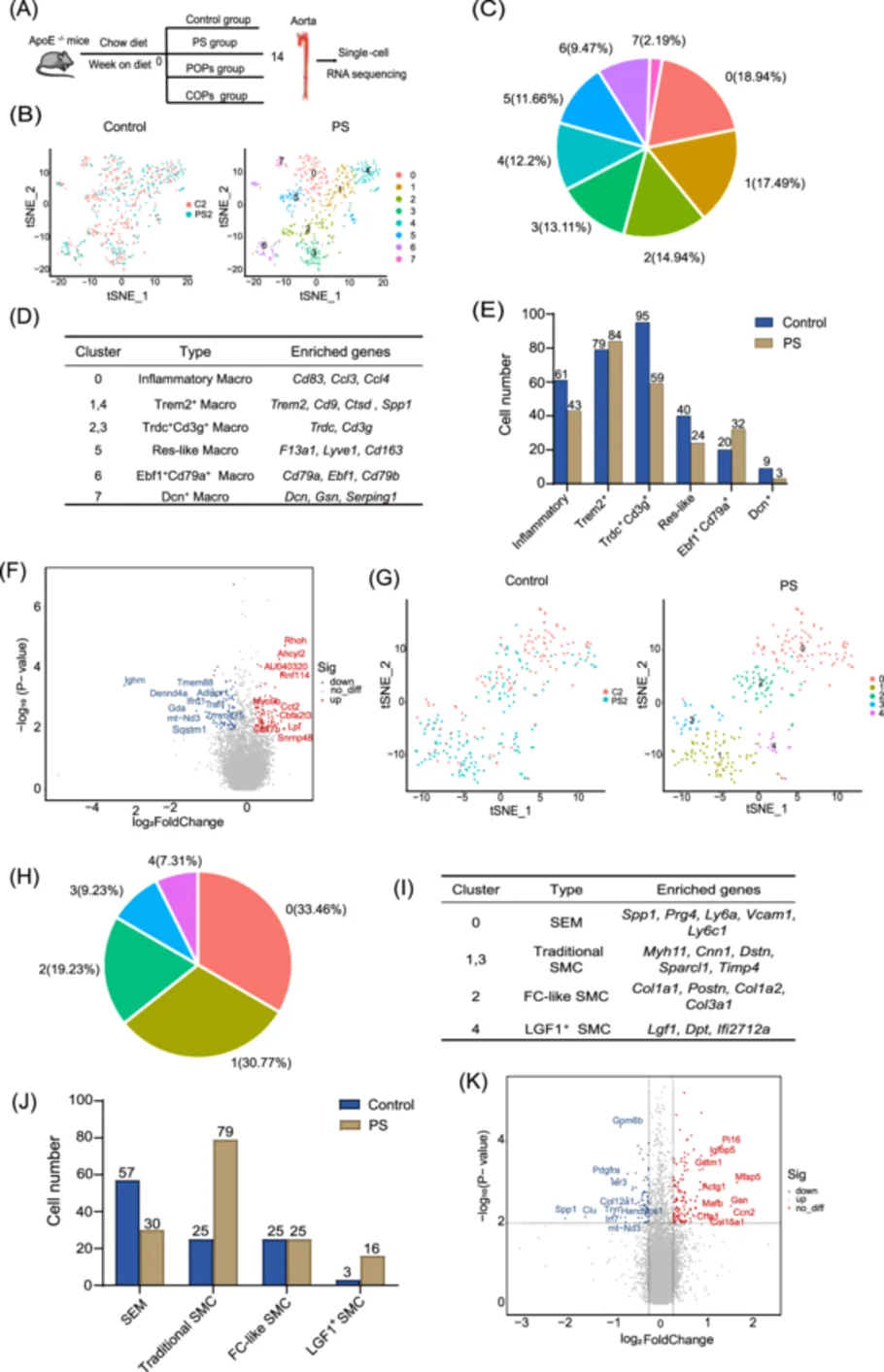
Heterogeneity of macrophage and smooth muscle cells (SMC) from *ApoE* ^−/−^ mice. (A) Flowchart of experimental design. For the control group, a dose of 0.2% cholesterol was contained in diets. The phytosterol (PS) diet, cholesterol oxidation products (COPs) diet, and phytosterol oxidation products (POPs) diet contained 0.02% PS, 0.02% COPs, and 0.02% POPs based on the control diet, respectively. After feeding for 14 weeks, the aorta was isolated for single‐cell RNA sequencing. (B) t‐SNE visualization of macrophage from the control group and PS group. (C) Fraction of macrophage subpopulations. (D) Cluster and macrophage subpopulation correspondence. (E) Effects of phytosterol on macrophage constitution. (F) Volcano plot expression between PS group and control group in inflammatory macrophage. (G) t‐SNE visualization of SMC from the control group and PS group. (H) Fraction of SMC subpopulations. (I) Cluster and SMC subpopulation correspondence. (J) Effects of phytosterol on SMC constitution. (K) Volcano plot expression between PS group and control group in SEM cells. SEM, SMC‐derived intermediate cells.

To evaluate the effects of PS on macrophages and SMCs, we performed subgroup analysis using cells from the control and PS‐treated groups (Supporting Information S1: Figure S6). Among macrophages, 8 distinct clusters were identified (Figure 1A–D, and Supporting Information S1: Table S2). Cluster 0 represented inflammatory macrophages characterized by high expression of *Cd83*, *Ccl3*, *Ccl4*, *Il‐1β*, *Nlrp3*, and *Cxcl12* [19], and was enriched in NF‐κB signaling and immune response pathways (Supporting Information S1: Figure S7). Notably, PS treatment significantly reduced the proportion of this pro‐inflammatory subset (Figure 1E). Cluster 1 and Cluster 4 were identified as *Trem2* ^*hi*^ microphages based on elevated expression of *Trem2*, *Lgals3*, *Cd9*, *Ctsb*, and *Ctsd*. These markers are associated with plaque regression and M2‐like anti‐inflammatory phenotypes [20, 21]. *Trem2* has been reported to provide protection against infections and glucose intolerance in mice, and its expression is linked to plaque stability in humans [22]. Cluster 5 was classified as resident‐like macrophages based on the expression of *F13a1*, *Lyve1*, *Ccl8*, and *Pf4* [19]. Cluster 6 was marked by high expression of *Ebf1* and *Cd79a* (Ebf1^hi^Cd79a^hi^), a subset associated with anti‐inflammatory properties and plaque regression [23]. Cluster 7, characterized by *Dcn*, *Gsn*, and *Col1a2* expression, appeared to represent a transitional population of both macrophages SMC. PS treatment markedly increased the proportion of *Trem2* ^*hi*^ and *Ebf1* ^*hi*^ *Cd79a* ^*hi*^ macrophages (Figure 1E), supporting its role in promoting anti‐inflammatory macrophage polarization.

Given the critical role of inflammatory macrophages in plaque progression [24], we further investigated the gene expression changes in Cluster 0 (Figure 1F). Genes significantly downregulated are shown in blue (log_2_FC < −0.26 and *p* < 0.01), while upregulated are shown in red (log_2_FC > 0.26 and *p* < 0.01). PS intervention suppressed key components of the NF‐κB pathway, including *Traf1* and *Clec7a*. *Traf1* is involved in regulation of NF‐κB and JNK signaling, while *Clec7a* encodes a pattern recognition receptor involved in TLR2‐mediated NF‐κB activation and pro‐inflammatory cytokine production [25, 26].

These findings suggest that PS mitigates vascular inflammation through inhibiting NF‐κB signaling, promoting a shift from pro‐inflammatory to anti‐inflammatory macrophage phenotypes, and thereby contributing to atheroprotection.

### Identification of smooth muscle cell heterogeneity induced by PS

We next examined the effects of PS on SMC heterogeneity and identified 5 distinct SMC subpopulations (Figure 1G–K, Supporting Information S1: Figure S8 and Supporting Information S1: Table S3). Cluster 1 exhibited enrichment for bone remodeling genes such as *Spp1* and *Prg4*, while Cluster 0 displayed upregulation of stem cell and macrophage‐related markers, including *Ly6a* and *Lgals3*. At the same time, canonical SMC markers (*Myh11*, *Acta2*, and *Tagln*) were downregulated, suggesting a loss of typical SMC phenotype. Consistent with Pan et al. [27], we classified Cluster 0 cells as SMC‐derived intermediate cells (SEMs), characterized by the expression of *Ly6a*, *Vcam1*, and *Ly6c1*, and possessing the potential for multilineage differentiation. Pathway enrichment analysis revealed that SEMs were associated with increased activity in extracellular matrix remodeling, endoplasmic reticulum processing, and actin cytoskeleton organization (Supporting Information S1: Figure S8). Cluster 2 was identified as fibrochondrocyte (FC)‐like SMCs, characterized by high expression of *Col1a1*, *Postn*, *Col1a2*, and *Col3a1*, along with downregulation of classical SMC markers such as *Myh11*. Clusters 1 and 3 were defined as classical contractile SMCs, due to their high expression of *Myh11*, *Acta2*, and *Tagln*. Cluster 4 exhibited high expression of genes related to cell proliferation and migration and was thus defined as *Lgf1* ^+^ proliferative subset.

SEM accounted for approximately 33.46% of the total SMC population (Figure 1H). PS treatment reduced the proportion of SEM cells while increasing the proportion of contractile SMCs (Figure 1J), suggesting that PS may inhibit the transition of SMCs into SEMs and limit their differentiation into macrophage‐like and FC‐like phenotypes. Differential gene expression analysis of SEMs revealed that PS treatment upregulated *Gpm6b* and *Pdgfra* expression (Figure 1K). *Gpm6b* has been implicated in SMC differentiation, while *Pdgfra*‐mediated signaling is crucial for differentiating embryonic stem cells into mature SMCs and maintaining SMC identity [28]. The upregulation of these genes suggests that PS may reinforce SMC stability and suppress phenotypic switching. Additionally, PS increased the expression of *Pi16* and *Igfbp5*, both of which have been associated with vascular homeostasis. While *Pi16* has been linked to cardiac hypertrophy and vascular fibrosis [29], its direct role in SEM biology remains unclear. Collectively, our findings suggest that PS may exert vascular protective effects by modulating SMC differentiation and stabilizing the contractile phenotype, thereby limiting pathological remodeling in AS.

### Stigmasterol inhibits ox‐LDL‐induced macrophage foam cell formation

**Figure 2:**
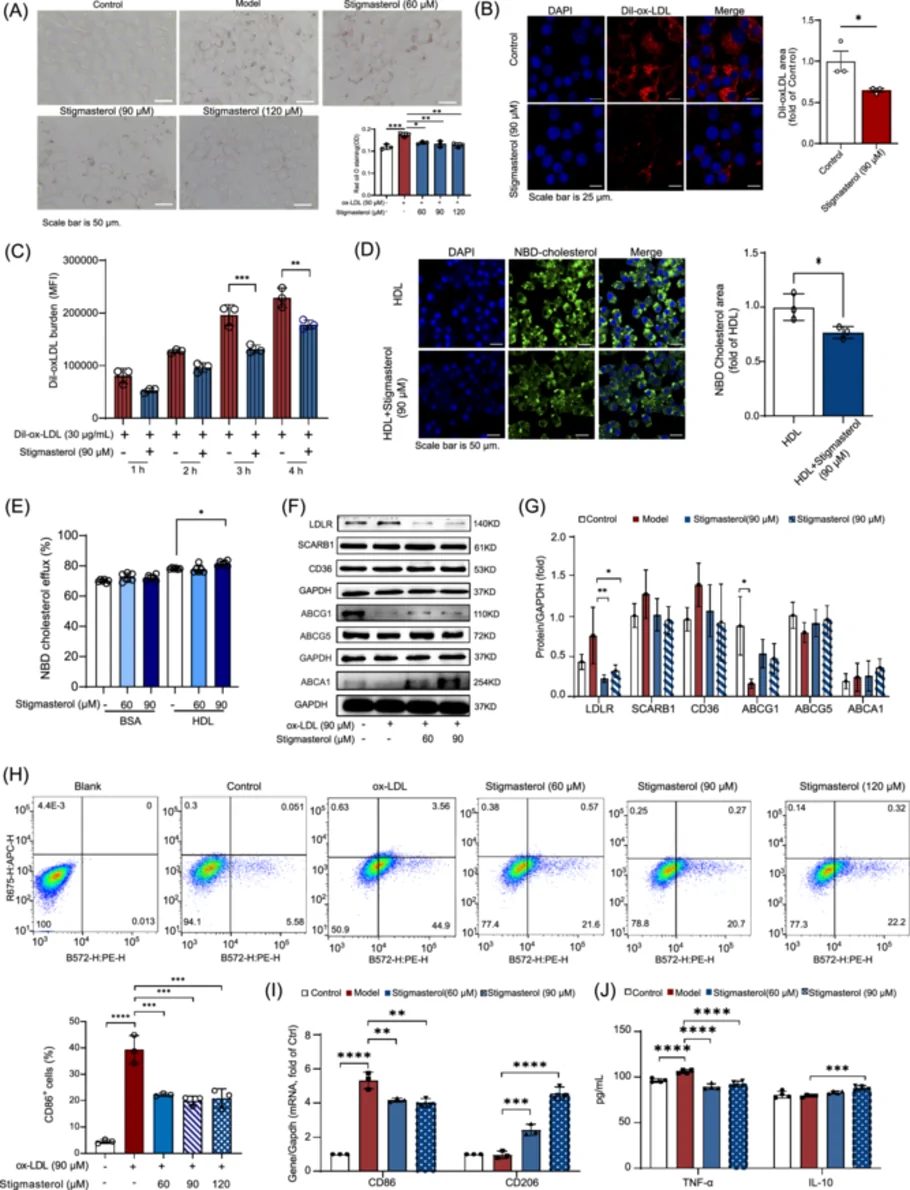
Effects of stigmasterol on cholesterol metabolism and phenotypic transition in ox‐LDL‐treated macrophages. (A) Cells were incubated with stigmasterol (60, 90, and 120 μM) for 6 h, then co‐incubated with ox‐LDL for 24 h. Morphologic change of foam cells stained by Oil Red O (ORO) and quantitation of lipid drops by extracting ORO dyes from the stained macrophages (magnification ×200). The scale bar in the figure is 50 μm. *n* = 3. (B) Cells were pretreated with stigmasterol for 6 h, then incubated with 10 μg/mL Dil‐ox‐LDL and stigmasterol for an additional 4 h. Dil‐LDL (red) fluorescence represents DiI‐LDL particles while blue fluorescence indicates nucleus. (magnification ×400). The scale bar in the figure is 25 μm. (C) The mean Dil‐ox‐LDL burden (mFI) was analyzed by flow cytometry. *n* = 3. (D) Cells were loaded with NBD‐cholesterol (5 μg/mL) (green) and stigmasterol (90 μM) for 12 h. Then incubated for 4 h with 0.2% BSA or HDL (50 μg/mL). Green fluorescence represents NBD‐cholesterol particles while blue fluorescence indicates the nucleus. (magnification ×200). The scale bar in the figure is 50 μm. (E) HDL‐mediated cholesterol efflux (%) was measured in macrophages treated with stigmasterol or control by using fluorescence plate reader. *n* = 6. (F) Expressions of LDLR, SCARB1, CD36, ABCG1, ABCG5, and ABCA1 after ox‐LDL or stigmasterol treatment (60 and 90 μM) were evaluated by western blot. (G) Qualification of LDLR, SCARB1, CD36, ABCG1, ABCG5 and ABCA1. *n* = 3. (H) Cells were incubated with stigmasterol for 6 h, then co‐incubated with ox‐LDL for 24 h. The percentage of CD86^+^ cells was analyzed by flow cytometry. *n* = 3. (I) *Cd86* and *Cd206* mRNA detection by q‐RT‐PCR treated with stigmasterol or ox‐LDL. *n* = 3. (J) Release of TNF‐α and IL‐10 treated with stigmasterol or ox‐LDL. *n* = 4. Multi‐group comparison was measured by one‐way ANOVA. The data are presented as the mean ± SD. *p < 0.05, **p < 0.01, ***p < 0.001. ANOVA, analysis of variance.

To compare the effects of β‐sitosterol, stigmasterol, and campesterol on lipid accumulation in macrophages, Oil Red O (ORO) staining was performed. Before evaluating lipid accumulation, we assessed the cytotoxicity of each compound using concentrations of 10, 60, 120, and 240 μM. As shown in Supporting Information S1: Figure S9, no cytotoxic effects were observed on macrophages at concentrations up to 120 μM for any of the three compounds. Based on these results, concentrations of 60, 90, and 120 μM were selected for subsequent experiments. We next assessed the effects of these PS on foam cell formation induced by ox‐LDL. Pretreatment with β‐sitosterol, stigmasterol, and campesterol significantly suppressed foam cell formation in a dose‐dependent manner. Following ORO staining, the lipid‐bound dye was extracted using isopropanol, and optical density (OD) was measured at 530 nm to quantify lipid content (Supporting Information S1: Figure S10). Both the staining and quantitative data demonstrated that stigmasterol exhibited the most potent inhibitory effect on lipid accumulation among the three compounds tested (Figure 2A). Based on these findings, stigmasterol was selected for further mechanistic analysis.

To determine whether stigmasterol affects ox‐LDL uptake, we measured intracellular fluorescence after treatment with Dioctadecyl‐3,3,3,3‐tetramethylin docarbocyanine‐labeled ox‐LDL (Dil‐ox‐LDL) at 1, 2, 3, and 4 h. Compared to the model group, stigmasterol significantly reduced the mean fluorescence intensity at both 3 and 4 h (*p *< 0.001), indicating it suppressed ox‐LDL uptake. Consistent with this data, fluorescence imaging revealed strong red fluorescence in the model group after 4 h, which was markedly diminished in stigmasterol‐treated cells (Figure 2B,C). Cholesterol efflux was then evaluated using NBD‐labeled cholesterol. Macrophages were pre‐loaded with 5 μg/mL of NBD‐cholesterol for 12 h. When treated with BSA lacking high‐density lipoprotein (HDL), stigmasterol did not significantly affect cholesterol efflux. However, in the presence of 50 μg/mL HDL, treatment with 90 μM stigmasterol significantly promoted cholesterol efflux compared to the control group (*p *< 0.05) (Figure 2D,E). We further examined gene expression related to cholesterol metabolism. Stigmasterol significantly reduced the expression of the low‐density lipoprotein receptor (LDLR) (*p* < 0.01) but did not alter the expression of major cholesterol efflux transporters ABCG1, ABCG5, and ABCA1 (Figure 2F,G). These results suggest that stigmasterol primarily reduces intracellular lipid accumulation by suppressing cholesterol uptake, rather than by promoting cholesterol efflux. In summary, stigmasterol exerts a strong lipid‐lowering effect in macrophages by inhibiting ox‐LDL uptake, highlighting its potential role in regulating macrophage lipid metabolism and attenuating foam cell formation.

### Stigmasterol inhibits inflammatory macrophages and modulates AMPK/NF‐κB/NLRP3 pathway

scRNA‐seq analysis revealed that PS treatment reduced the proportion of inflammatory macrophages. As CD86 is a marker of pro‐inflammatory macrophage polarization, we examined the proportion of CD86^+^ cells by flow cytometry. Following ox‐LDL stimulation, the proportion of CD86^+^ cells increased significantly to 44.9% (Figure 2H). Treatment with 60 and 90 μM stigmasterol reduced this proportion to 21.6% and 20.7%, respectively (Figure 2H). In parallel, *Cd86* mRNA levels were significantly downregulated (*p* < 0.01), while the expression of *Cd206*, a marker of anti‐inflammatory macrophages, was significantly upregulated (*p* < 0.0001) in stigmasterol‐treated macrophages (Figure 2I). Additionally, stigmasterol markedly suppressed TNF‐α secretion (*p *< 0.0001), a key pro‐inflammatory cytokine (Figure 2J). Consistently, these findings suggest that stigmasterol mitigates inflammatory responses and inhibits inflammatory macrophage polarization.

**Figure 3:**
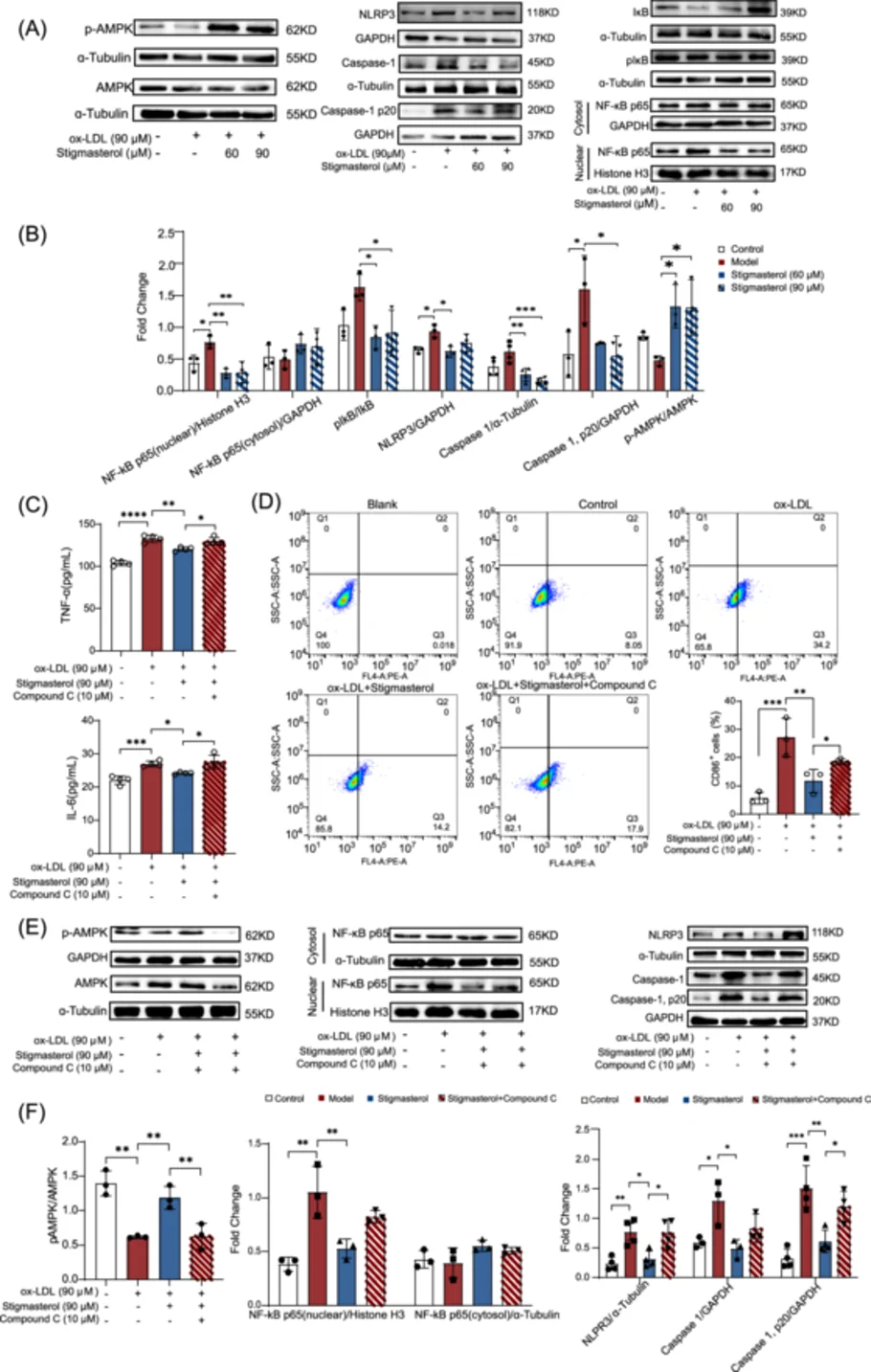
Stigmasterol inhibits macrophage phenotypic transition to pro‐inflammatory type by influencing the AMPK/NF‐κB/NLRP3 pathway. Cells were incubated with stigmasterol for 6 h, then co‐incubated with ox‐LDL or Compound C (10 μM) for 24 h. (A) Expressions of IκB, pIκB, NF‐κB p65, AMPK, p‐AMPK, NLRP3, Caspase‐1, and Caspase‐1 p20 in the protein of macrophages after ox‐LDL or stigmasterol treatment were evaluated by Western blot. (B) Quantification of NF‐κB (cytosol), NF‐κB (nuclear), pIκB/IκB, NLRP3, p‐AMPK/AMPK, Caspase‐1 p20 and Caspase‐1. *n* = 3. (C) Expression of TNF‐α and IL‐6 was evaluated by using ELISA kit. *n* = 4. (D) The percentage of CD86^+^ cells in the control group, model group, stigmasterol group, and compound C group was analyzed by flow cytometry. *n* = 3. (E) Expression of AMPK, p‐AMPK, NF‐κB, NLRP3, Caspase‐1, and Caspase‐1 p20 in the control group, model group, stigmasterol group, and compound C group was evaluated by western blot. (F) Quantification of p‐AMPK/AMPK, NF‐κB (cytosol), NF‐κB (nuclear), NLRP3, Caspase‐1, and Caspase‐1 p20 after Compound C treatment. *n* = 3. Multi‐group comparison was measured by one‐way ANOVA. The data are presented as the mean ± SD. *p < 0.05, **p < 0.01, ***p < 0.001, ***p < 0.0001. ANOVA, analysis of variance; ELISA, enzyme‐linked immunosorbent assay.

Given that our scRNA‐seq data indicated PS may regulate NF‐κB signaling, we further examined the expression of proteins involved in this pathway (Figure 3A,B). Ox‐LDL treatment increased the phosphorylation of IκB and nuclear NF‐κB levels (*p* < 0.05), indicating pathway activation. Stigmasterol treatment at both 60 and 90 μM significantly inhibited NF‐κB activation. Notably, the AMPK/NF‐κB axis is closely linked to NLRP3 inflammasome activity, a key driver of chronic vascular inflammation. Since AMPK activation is known to suppress both NF‐κB signaling and NLRP3 inflammasome activation, we assessed the expression of key components within these pathways. Ox‐LDL exposure led to increased expression of NLRP3 and cleaved Caspase‐1 (Caspase‐1 p20) (*p* < 0.05), while stigmasterol significantly inhibited their expression. Additionally, ox‐LDL reduced AMPK phosphorylation (p‐AMPK) levels, whereas stigmasterol treatment significantly restored p‐AMPK levels in a dose‐dependent manner (*p* < 0.05) (Figure 3A,B). Together, these results suggest that stigmasterol activates the AMPK signaling and concurrently inhibits NF‐κB and NLRP3 inflammasome pathways.

To determine whether AMPK activation is required for the anti‐inflammatory effects of stigmasterol, we utilized Compound C, an AMPK inhibitor, to block AMPK signaling. Cells were divided into 4 groups: control, ox‐LDL (90 μg/mL), ox‐LDL + stigmasterol (90 μM), and ox‐LDL + stigmasterol + compound C (10 μM). The stigmasterol‐mediated suppression of TNF‐α and IL‐6 secretion (*p* < 0.05), as well as the reduction in CD86^+^ macrophages (*p *< 0.05), was significantly reversed after Compound C treatment (Figure 3C,D). Additionally, co‐treatment with stigmasterol and Compound C significantly reduced AMPK phosphorylation (*p* < 0.01), and the inhibitory effect of stigmasterol on the NF‐κB/NLRP3 pathway was significantly attenuated (*p *< 0.01) (Figure 3E,F). These findings confirm that stigmasterol attenuates ox‐LDL‐induced macrophage polarization through AMPK signaling pathway.

### Stigmasterol inhibits SMC‐derived foam cell formation and suppresses CD68 expression

**Figure 4:**
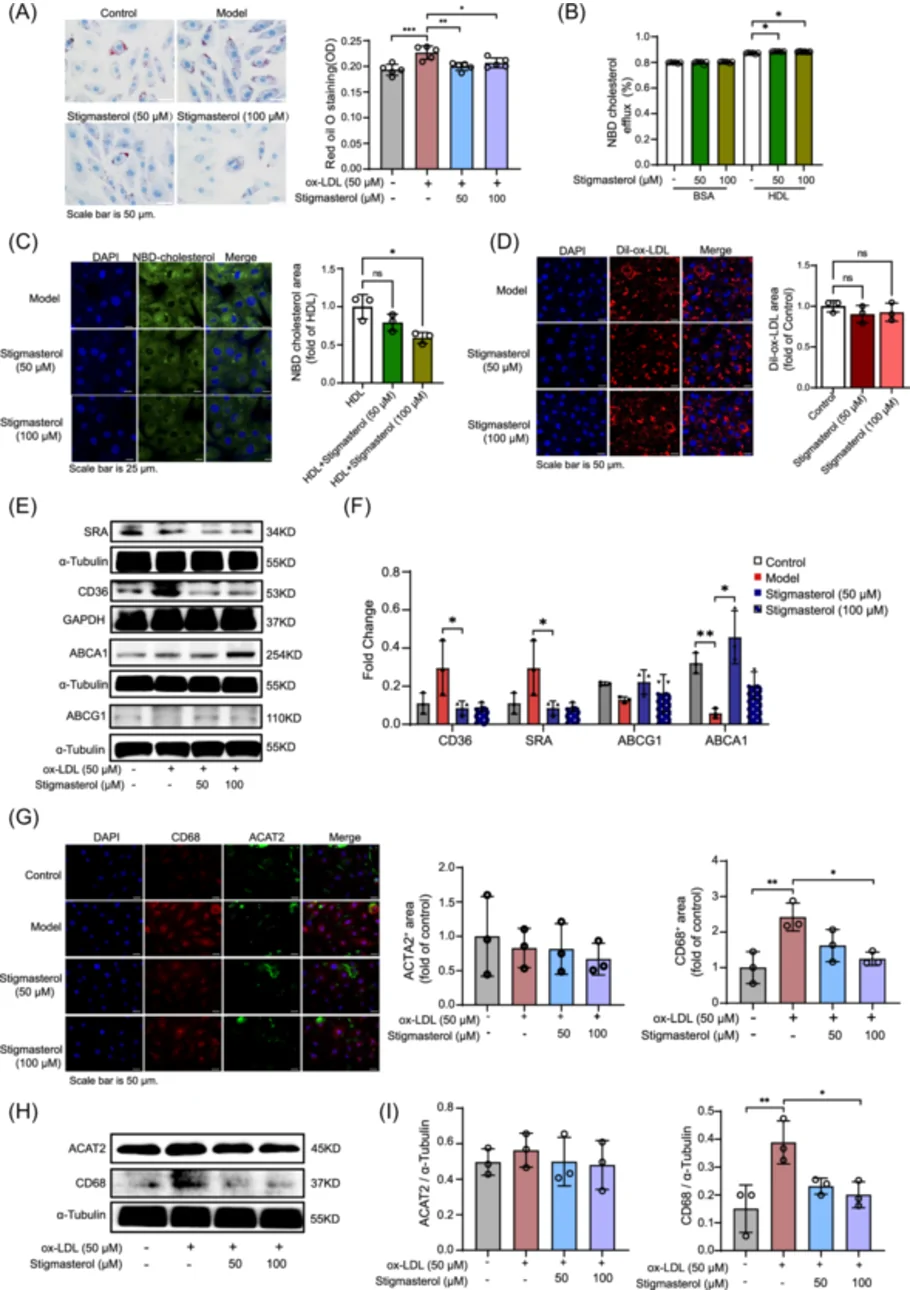
Effects of stigmasterol on lipid metabolism and phenotype change in ox‐LDL‐treated SMCs. (A) Cells were incubated with stigmasterol (50 and 100 μM) for 6 h, then co‐incubated with ox‐LDL for 24 h. Morphologic change of foam cells stained by ORO and quantitation of lipid drops by extracting ORO dyes from the stained SMCs (magnification ×200). The scale bar in the figure is 50 μm. *n* = 5. (B) Cells were loaded with NBD‐cholesterol (5 μg/mL) (green) and stigmasterol (50 or 100 μM) for 12 h. Then incubated for 4 h with 0.2% BSA or HDL (50 μg/mL). HDL‐mediated cholesterol efflux (%) was measured by using fluorescence microplate reader. *n* = 5. (C) Representative fluorescence image of SMCs cultured with NBD‐cholesterol (green) and stigmasterol after 4 h of HDL incubation (magnification ×400). The scale bar in the figure is 25 μm. (D) Cells were pretreated with stigmasterol for 6 h, then incubated with 10 μg/mL Dil‐ox‐LDL and stigmasterol for an additional 4 h. Dil‐LDL (red) fluorescence represents Dil‐ox‐LDL particles while blue fluorescence indicates nucleus (magnification ×200). The scale bar in the figure is 50 μm. (E) Expression of SRA, CD36, ABCA1, and ABCG1 after ox‐LDL or stigmasterol treatment was evaluated by western blot. (F) Quantification of SRA, CD36, ABCA1, and ABCG1. *n* = 3. (G) Immunofluorescent staining with CD68 (red) and ACTA2 (green) antibodies in SMCs after stigmasterol treatment (magnification ×200). The scale bar in the figure is 50 μm. *n* = 3. (H) CD68 and α‐SMA in SMCs were evaluated by Western blot after stigmasterol treatment. (I) Quantification of CD68 and α‐SMA. *n* = 3. Multi‐group comparison was measured by one‐way ANOVA. The data are presented as the mean ± SD. *p < 0.05, **p < 0.01, ***p < 0.001. ANOVA, analysis of variance; HDL, high‐density lipoprotein; SMC, smooth muscle cells.

To explore the potential of stigmasterol to reduce lipid accumulation in SMC‐derived foam cells, we first evaluated its cytotoxicity in smooth muscle A7r5 cells. Cells were treated with stigmasterol at concentrations ranging from 25 to 200 μM for 24 h. Cell viability assays indicated that stigmasterol was noncytotoxic up to 200 μM (Supporting Information S1: Figure S11). Based on these findings, 50 and 100 μM were selected for subsequent experiments. ORO staining revealed a significant reduction in lipid accumulation in SMCs treated with stigmasterol compared to the control group (*p *< 0.01) (Figure 4A). Consistent with its effects in macrophages, stigmasterol enhanced cholesterol efflux in SMCs (*p *< 0.05) (Figure 4B,C). However, 50 μM stigmasterol did not significantly affect the uptake of Dil‐labeled ox‐LDL (Figure 4D), while western blot results confirmed the upregulation of ABCA1 expression (Figure 4E,F). These results suggest that stigmasterol promotes cholesterol flux primarily by enhancing efflux, rather than by inhibiting ox‐LDL uptake in SMCs.

Given that scRNA‐seq data indicated phenotypic plasticity in SMCs, we next used immunofluorescence to assess the expression of ACTA2 and CD68, markers of contractile and macrophage‐like SMC phenotypes, respectively (Figure 4G). ACTA2, also known as α‐smooth muscle actin (α‐SMA), showed no significant difference between groups, indicating that the contractile identity of SMCs was maintained. In contrast, CD68 was significantly upregulated in the ox‐LDL–treated model group (*p* < 0.01) and reduced by treatment with 100 μM stigmasterol (*p* < 0.05). These observations were further validated by Western blot analysis (Figure 4H,I). Together, these findings support that SMCs can undergo phenotypic switching toward a macrophage‐like foam cell state in response to lipid stress. Stigmasterol inhibits this trans‐differentiation process by reducing CD68 expression and enhancing cholesterol efflux.

### Stigmasterol suppresses CD86^+^ macrophage and F4/80^+^α‐SMA^+^ cells in *ApoE* ^−/−^ mice

**Figure 5:**
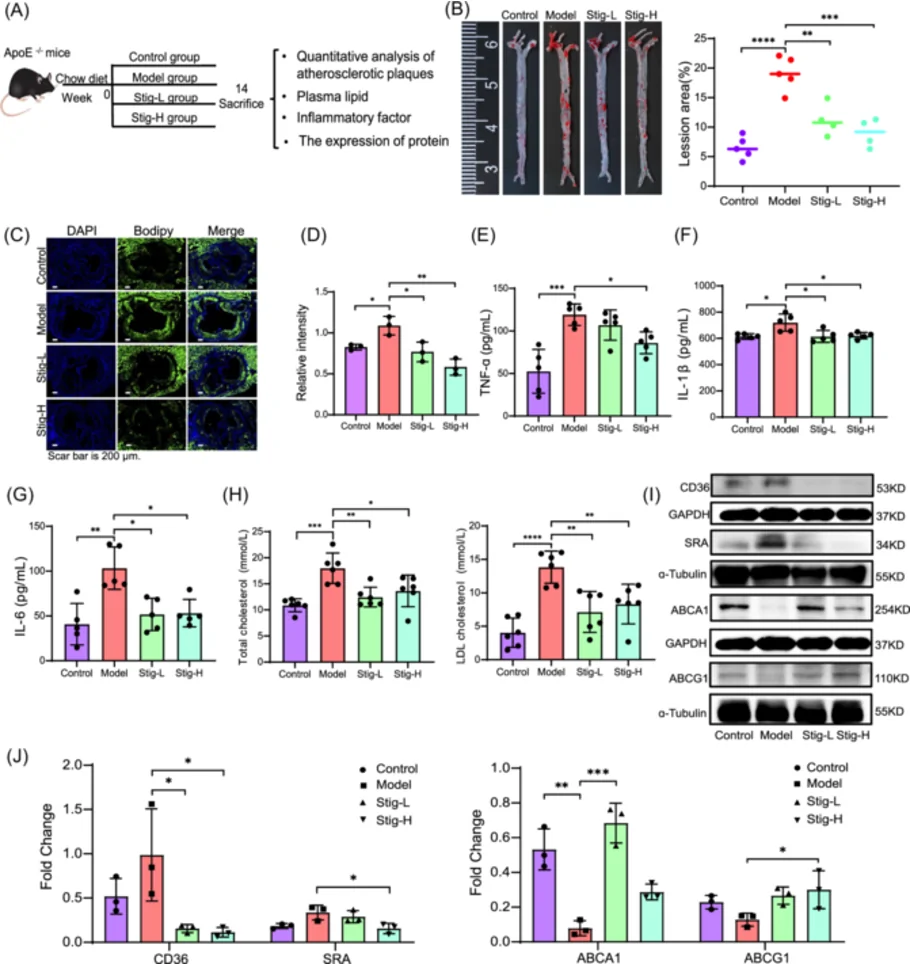
Effects of stigmasterol on atherosclerosis plaque and lipid levels. (A) Flowchart of experimental design. 8‐week‐old mice were fed on control, model (0.5% cholesterol), low‐dose stigmasterol group (Stig‐L), and high‐dose stigmasterol group (Stig‐H) for 14 weeks. The Stig‐L and Stig‐H groups were supplemented with 1.5 and 3 g/kg of stigmasterol, respectively, based on the model feed. (B) Lipid‐stained images and quantification of the whole aorta by Oil red O. *n* = 4 or 5. (C) Bodipy staining of aortic root sections (magnification ×4.5). The scale bar in the figure is 200 μm. (D) The relative intensity of Bodipy in aortic root sections. *n* = 3. (E–G) Levels of inflammatory factors in plasma on the 14th week. *n* = 5. (H) Total cholesterol and LDL cholesterol levels in plasma on the 14th week. *n* = 6. (I) Expression of SRA, CD36, ABCA1, and ABCG1 in the aorta from different groups was evaluated by western blot. (J) Quantification of SRA, CD36, ABCA1, and ABCG1. *n* = 3. Multi‐group comparison was measured by one‐way ANOVA. The data are presented as the mean ± SD. *p < 0.05, **p < 0.01, ***p < 0.001. ANOVA, analysis of variance.

**Figure 6:**
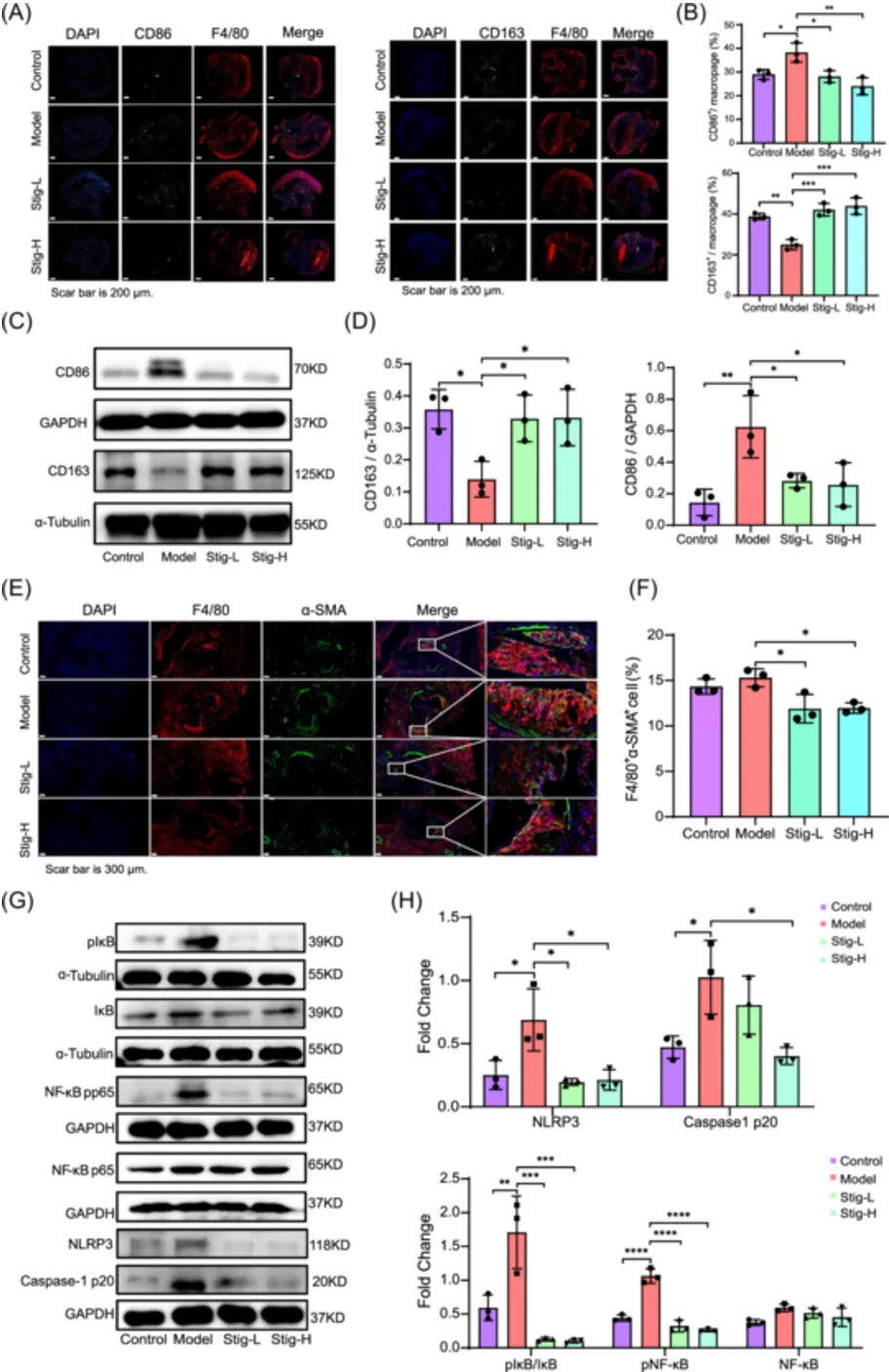
Effects of stigmasterol on aortic macrophage and SMC phenotype in *ApoE* ^−/−^ mice. (A) Images of immunofluorescence staining for CD163 (green), CD86 (green), and F4/80 (red) in the aorta. The scale bar in the figure is 200 μm. (B) Percentage of CD86^+^ and CD163^+^ macrophages. *n* = 3. (C) Expression of CD86 and CD163 in the aorta was revealed by western blot. (D) Qualification of CD86^+^ and CD163^+^. *n* = 3. (E) Images of immunofluorescence staining for α‐SMA (green) and F4/80 (red) in the aorta. The scale bar in the figure is 300 μm, and the image in the right frame is a ×400 magnification of the point pointed. *n* = 3. (F) Percentage of F4/80^+^α‐SMA^+^ cells. *n* = 3. (G) Expression of NF‐κB p65, NF‐κB pp65, NLRP3, and Caspase‐1 p20 in the aorta by western blot. (H) Qualification of NF‐κB p65, NF‐κB pp65, NLRP3, and Caspase‐1 p20. *n* = 3. Multi‐group comparison was measured by one‐way ANOVA. The data are presented as the mean ± SD. *p < 0.05, **p < 0.01, ***p < 0.001, ****p < 0.0001. ANOVA, analysis of variance.

To validate the impact of stigmasterol on the phenotypes of macrophages and SMCs in vivo, we established a mouse model utilizing *ApoE* ^−/−^ mice (Figure 5A). Mice were fed either a control diet, a western diet (model group), or a western diet supplemented with low‐dose (1.5 g/kg) or high‐dose (3.0 g/kg) stigmasterol from 7 to 21 weeks of age. Over the 14 weeks, neither dose of stigmasterol significantly affected body weight or food intake (Supporting Information S1: Figure S12). Histological analysis revealed that stigmasterol significantly reduced atherosclerotic plaque area (*p* < 0.001) and foam cell formation (*p *< 0.01) compared to the model group (Figure 5B–D). Biochemical assays further demonstrate that stigmasterol markedly lowered inflammatory markers (Figure 5E–G) total cholesterol (TC), and LDL‐C levels (*p *< 0.01) (Figure 5H). To further evaluate the effects on lipid metabolism, we performed western blot analysis on aortic tissues. Stigmasterol significantly downregulated the expression of CD36 and SRA (*p* < 0.05), while significantly upregulating the cholesterol efflux transporter ABCA1 (*p* < 0.001) and ABCG1 (*p *< 0.05), suggesting enhanced cholesterol efflux (Figure 5I,J). Immunofluorescence analysis showed that stigmasterol significantly decreased the number of proinflammatory CD86⁺ macrophages (*p* < 0.01), while increasing the proportion of anti‐inflammatory CD163⁺ macrophages (*p *< 0.001) (Figure 6A,B). These changes in macrophage polarization were corroborated by western blot analysis (Figure 6C,D). Furthermore, stigmasterol reduced the proportion of F4/80⁺α‐SMA⁺ cells, a population indicative of macrophage‐like SMCs (*p *< 0.05) (Figure 6E,F). Additionally, western blot analysis demonstrated that stigmasterol inhibited the activation of NF‐κB/NLRP3 pathway (*p *< 0.05) (Figure 6G,H). Collectively, these findings suggest that stigmasterol suppresses SMC‐to‐macrophage trans‐differentiation and attenuates inflammatory macrophage polarization in vivo, highlighting the therapeutic potential of stigmasterol in the treatment of AS.

## 3 DISCUSSION

PS, structurally similar to cholesterol, reduces plasma cholesterol levels by competitively inhibiting cholesterol intestinal absorption. Due to active excretion, their systemic absorption is minimal, and most PS are eliminated via bile. Once in circulation, PS are incorporated into lipoproteins, mainly HDL, undergo limited hepatic metabolism, and are rapidly excreted, unlike cholesterol, which is efficiently converted into steroid hormones and bile acids. Notably, PS can also be oxidized into phytosterol oxidation products, which may have distinct biological effects. While the cholesterol‐lowering effects of PS are well established, their role in foam cell formation, particularly at the single‐cell level, remains unclear. Building on prior work from our lab showing that PS improves hepatic lipid metabolism [18], we used aortic tissues from that study to examine the impact of PS on macrophage and SMC heterogeneity using scRNA‐seq. We identified 8 macrophage subpopulations, with inflammatory macrophages as the most abundant, enriched for genes involved in NF‐κB signaling. PS treatment reduced the proportion of this pro‐inflammatory subset and downregulated NF‐κB–associated genes. In SMCs, five distinct subtypes were identified, including a SEM phenotype, which shares features with macrophages and fibrochondrocytes and reflects a loss of contractile identity. PS decreased the proportion of SEM cells and promoted a contractile phenotype, suggesting PS may stabilize SMC identity and inhibit foam cell formation. Based on these findings, we conducted in vitro experiments using RAW264.7 macrophages and A7r5 SMCs to further explore the cellular effects of PS. Among the compounds tested, stigmasterol showed the strongest ability to inhibit lipid droplet accumulation, outperforming β‐sitosterol and campesterol. Prior research showed that stigmasterol enhances cholesterol efflux in THP‐1 macrophages by regulating ABCA1 and ABCG1 [30]. Consistent with this, our results demonstrated that stigmasterol promotes NBD‐cholesterol efflux, reduces Dil‐oxLDL uptake, and modulates LDLR1 and ABCG1 expression.

Since inflammation drives foam cell formation, we also assessed the effect of stigmasterol on macrophage polarization. Stigmasterol reduced the proportion of ox‐LDL‐induced CD86^+^ inflammatory macrophages, decreased CD86 receptor expression, and suppressed pro‐inflammatory cytokines, including TNF‐α and IL‐6. Mechanistically, Stigmasterol inhibited the activation of NF‐κB pathway and the NLRP3 inflammasome.

AMPK is a key regulator of energy metabolism and inflammation, known to suppress NF‐κB signaling [31]. Previous studies have reported that β‐sitosterol stimulates AMPK phosphorylation and exerts anticancer effects [32], and that phytosterol esters of α‐linolenic acid protect against nonalcoholic fatty liver disease via AMPK signaling [33]. These findings collectively highlight AMPK as a potential therapeutic target. In our study, the inhibition of AMPK reversed the effects of stigmasterol on the NF‐κB/NLRP3 axis and CD86 expression, underscoring the critical role of AMPK in stigmasterol‐mediated macrophage polarization. Furthermore, in *ApoE* ^−/−^ mice, stigmasterol decreased the proportion of proinflammatory CD86^+^ macrophages and increased anti‐inflammatory CD163^+^ macrophages. This shift in macrophage subtype was accompanied by suppression of key proteins in the NF‐κB/NLRP3 pathway, reinforcing the anti‐inflammatory role of stigmasterol in vivo.

SMCs constitute over 50% of foam cells within arterial plaques [34]. Cholesterol accumulation, particularly in the context of ABCA1 dysfunction, can drive SMCs toward a synthetic and proliferative phenotype that promotes AS progression. Studies have shown that ABCA1 expression is lower in human coronary endometrial SMCs compared to endometrial macrophages, possibly making them more prone to foam cell formation [35]. Notably, our findings demonstrate that stigmasterol attenuates lipid accumulation in SMCs and facilitates cholesterol efflux by upregulating ABCA1. Consequently, ABCA1 appears to be a critical target of stigmasterol in SMC regulation. However, stigmasterol had no significant effect on ABCG1 expression, suggesting that its impact on lipid metabolism occurs through distinct mechanisms in macrophages versus SMCs.

Given previous evidence that SMC‐derived foam cells often express macrophage markers [36, 37], we investigated whether stigmasterol affects macrophage‐like marker expression in SMCs. We found that stigmasterol suppressed surface expression of CD68 in A7r5 cells, thereby impeding ox‐LDL‐induced SMC‐to‐macrophage trans‐differentiation. These findings align with our observations, where stigmasterol reduced the proportion of aortic F4/80^+^α‐SMA^+^ cells. Its inhibitory effect on F4/80 expression further supports the notion that stigmasterol mitigates SMC plasticity and prevents their conversion into macrophage‐like foam cells. Supporting this concept, a previous study reported that all‐trans retinoic acid, an anticancer drug, can promote SMC transition to a synthetic phenotype, decrease atherosclerotic plaque formation, and enhance fibrous cap stability [27]. These observations emphasize that targeting SMC phenotypic modulation is a promising strategy for AS therapy. Collectively, our findings underscore the multifaceted anti‐inflammatory and lipid‐lowering properties of stigmasterol. By modulating macrophage polarization, promoting cholesterol efflux, and inhibiting SMC phenotypic switching, stigmasterol emerges as a promising therapeutic candidate for AS.

Despite these insights, several limitations must be acknowledged. First, our single‐cell analysis was conducted using a phytosterol mixture (β‐sitosterol, campestral, and stigmasterol), which provided a comprehensive overview but may obscure the unique cellular effects of individual compounds. While stigmasterol exhibited superior lipid‐lowering effects in in vitro and in vivo models, future single‐cell studies focusing specifically on stigmasterol are warranted. Second, the observed changes in cell composition following PS treatment resemble those in models of plaque progression and regression [19, 27], suggesting that some of the beneficial effects may be mediated not only by the direct impact of PS on macrophage and SMC function, but also indirectly through their plasma lipid‐lowering properties. To distinguish these contributions, future studies in wild‐type mice would be valuable. Lastly, the number of cells within certain subpopulations was relatively low (Supporting Information S1: Tables S2 and S3). Increasing sample size and refining cell‐sorting or enrichment techniques would enhance resolution and improve the robustness of cell‐type‐specific analysis in future studies.

## 4 CONCLUSION

In summary, our study demonstrates that phytosterol supplementation effectively inhibits foam cell formation and lipid accumulation in both *ApoE* ^*−/−*^ mice and in vitro cell models. scRNA‐seq revealed the phytosterol mixtures exert beneficial effects by reducing the proportions of inflammatory macrophages and SMC‐derived intermediate phenotypes. Mechanistically, we identified stigmasterol as a key bioactive component that regulates macrophage polarization by attenuating inflammation via inhibition of the NF‐κB signaling pathway and suppression of NLRP3 inflammasome activation. These findings provide important mechanistic insights into the cardiovascular protective effects of PS and support their potential as therapeutic agents for the prevention and treatment of AS.

## 5 METHODS

### scRNA‐seq and data analysis

The animal experiment procedures and dosing details are based on the study by Wang et al. [18]. The composition of phytosterol mixture (β‐sitosterol, campesterol, and stigmasterol), COPs, and POPs used in the diets is detailed in Supporting Information S1: Table S1. 8‐week‐old male *ApoE* ^−/−^ mice were randomly assigned to four groups (*n* = 10 per group). The control group received a standard western diet (0.2% (w/w) cholesterol) (TD.88137, Nantong Trophic Animal Feed High‐Tech Co. Ltd.). The remaining three groups received the same diet supplemented with 0.2 g kg^−1^ of PS, COPs, or POPs for 14 weeks. This dosage corresponds to an estimated human equivalent dose of 2.77 mg kg^−1^ body weight per day.

At the end of the feeding period, aortic tissues were harvested, and fat was carefully removed. The tissues were then minced and enzymatically digested at 37°C with constant shaking until fully dissociated. The resulting cell suspension was filtered through a 100 μm cell strainer and centrifuged at 3000 rpm for 5 min at 4°C. The supernatant was discarded, and the cell pellet was resuspended in 100 μL of PBS containing 0.04% BSA and incubated with 1 mL of red blood cell lysis buffer at room temperature for 2–10 min. Subsequently, cells were centrifuged at 3000 rpm at room temperature for 5 min and resuspended in PBS. To stain for viability, cells were incubated with LIVE/DEAD™ Fixable Near‐IR Dead Cell Stain Kit and Hoechst 33342 for 20 min. Nucleated cells (Hoechst+/APC/Cy7−) were sorted using a flow cytometer and resuspended in PBS containing 0.04% BSA. Dead cells were removed using a dead cell removal kit according to the manufacturer's instruction. After a final centrifugation step (3000 rpm, 3 min, 4°C), cell viability was assessed by trypan blue exclusion and confirmed to be > 85%. Viable single‐cell suspensions were counted using a hemocytometer or Countess II automated cell counter and adjusted to a concentration of 700–1200 cells/μL. Cells were loaded onto the 10× Genomics Chromium Controller Single‐Cell 3′ kit (V3) according to the manufacturer's instructions to capture single cells. Subsequent steps, including cDNA synthesis and library construction, were performed per standard procedures. Libraries were sequenced on an Illumina NovaSeq. 6000 platform (LC‐Biotechnology Co. Ltd.) using paired‐end multiplexing operation (150 bp), targeting a minimum sequencing depth of 60,000 reads per cell. Raw base call (BCL) files were converted to FASTQ format using Illumina's bcl2 fastq software. For data analysis, the FASQ files were processed using Seurat (version 3.1.1) as follows: (1) Gene expression values were normalized using the NormalizeData function. (2) principal component analysis (PCA) and the top 10 ranked principal components were used for subsequent clustering and visualization with t‐distributed stochastic neighbor embedding (t‐SNE). (3) Marker genes for each cluster were determined using the FindAllMarkers function with default parameters. (4) Differentially expressed genes (DEGs) between experimental groups were identified using the FindAllMarkers function, applying a significance threshold of *p* < 0.01. DEGs with a log2 fold change >0.26 were further subjected to gene ontology (GO) enrichment analysis using DAVID. The downstream analysis of single‐cell RNA sequencing was performed using the cloud platform of LC‐Biotechnology Co. Ltd. (https://www.omicstudio.cn/home).

### Animal study design

8‐week‐old male *ApoE* ^−/−^ mice (GemPharmatech Co. Ltd.) were randomly divided into four groups. Mice were fed one of the following diets for 14 weeks: a low‐fat and low‐sugar control diet (TP26352, Hayek Western diet model feed), a Western diet containing 0.5% cholesterol (TP26304, model group), or a western diet supplemented with stigmasterol at either a low‐dose (1.5 g/kg, Stig‐L group) or a high‐dose (3 g/kg, Stig‐H group). All diets were manufactured by Nantong Trophic Animal Feed High‐Tech Co. Ltd. Food intake and body weight were recorded weekly.

After 14 weeks of dietary intervention, mice were killed for further analysis. The Aortic plaque area was assessed by ORO staining of the entire aorta. Blood was collected to measure serum total cholesterol and LDL cholesterol levels using commercial kits (Infinity, Thermo Scientific), as well as cytokines including TNF‐α, IL‐1β, and IL‐6 (Meimian). The aorta was dissected under a stereomicroscope. Fat tissue was carefully removed, and the adventitia was retained. The aorta was cut at the bifurcation of the bilateral arteries. The whole aortic root tissues were lysed to extract proteins for western blot analysis of target proteins. Additionally, cross‐sections of the aortic root were prepared for BODIPY 493/503 staining and immunofluorescence to evaluate lipid deposition and cellular markers.

### Cell culture and treatment

RAW264.7 cells (Shanghai EK‐Bioscience Biotechnology Co. Ltd.) and A7r5 (Wuhan Procell Biotechnology Co. Ltd.) cells were cultured in complete high‐glucose DMEM (Gibco) supplemented with 10% fetal bovine serum and 1% penicillin‐streptomycin. Cell culture dishes and plates were obtained from NEST Biotechnology Co. Ltd. Cells were pre‐incubated with stigmasterol for 6 h, followed by co‐incubation with ox‐LDL (YB‐0020; Yiyuan Biotech) or the AMPK inhibitor Compound C (10 μM; Selleck) for an additional 24 h. Cell viability assay was conducted using Cell Counting Kit‐8 (CCK‐8) (Beyotime Biotechnology). After treatment, cell supernatants were collected and the concentrations of inflammatory cytokines were measured using enzyme‐linked immunosorbent assay (ELISA) kits (Meimian).

### Oil red O staining

Cells were stained by Oil red O kit (G1262; Solabiro) according to the manufacturer's instruction. Stained lipid droplets were imaged using a bright‐light microscope to assess intracellular accumulation. For quantitative analysis, the dye retained in lipid droplets was extracted with isopropanol and the absorbance was measured at 520 nm using a microplate reader.

### Western blot

Protein lysates were extracted from cells or tissue samples using RIPA buffer (Shandong Sparkjade Biotechnology Co. Ltd.). Protein samples were separated by SDS‐PAGE and transferred onto PVDF membrane. Following transfer, membranes were blocked and incubated overnight at 4°C with primary antibodies. The primary antibodies used included: CD86 (DF6332; Affinity), CD163 (DF8235; Affinity), a‐SMA (AF1032; Affinity), CD68 (DF7518; Affinity), p‐AMPK (AF3423; Affinity), AMPK (AF6423; Affinity) IκB (AF5002; Affinity), pIκb (AF2002; Affinity), p‐NF‐κB (AF3219; Affinity), NLRP3 (DF15549; Affinity), Caspase 1 (AF5418; Affinity), Caspase 1 p20 (AF4005; Affinity), CD36 (DF13262; Affinity), SRA(DF14421, Affinity), LDLR (DF7696, Affinity), ABCA1 (DF8233), ABCG1 (DF8233, Affinity), ABCG5 (DF8401; Affinity), SCARB1 (DF6479; Affinity). Following primary antibody incubation, membranes were incubated with horseradish peroxidase (HRP)‐conjugated secondary antibody (S0001; Affinity). Protein bands were visualized using enhanced chemiluminescence detection reagents (ECL, Biosharp BL523A).

### Quantitative Real‐Time PCR (qRT‐PCR)

Total RNA was isolated using an RNA isolation kit (G3013; Servicebio), and RNA concentration and purity were assessed using a Nanodrop2000 spectrophotometer. cDNA was synthesized from total RNA using a Thermal Cycler (A37834; Thermo Scientific). qRT‐PCR was performed using the FastStart Universal SYBR Green Master (Rox) (Roche). The primers sequences used were as follows: *Cd86*, forward: TGACCGTTGTGTGTGTTCTGGA, reverse: TCTCTGTCAGCGTTACTATCCCG; *Cd206*, forward: CTCTGTCATCCCTGTCTCTGTTC, reverse: TGCCCTTGATTCCAAAGAGTGT; *Gapdh*, forward: CCTCGTCCCGTAGACAAAATG, reverse: TGAGGTCAATGAAGGGGTCGT.

### Flow cytometry

Cell suspensions were incubated with PE‐conjugated anti‐CD86 (x12‐0862‐82, 0.2 μg/mL; Thermo Fisher Scientific) for 20 min at 4°C. Samples were washed with PBS and analyzed by CytoFLEX LX flow cytometer (Beckman Coulter).

### Dil‐ox‐LDL uptake

Cells were seeded on confocal dishes, and pretreated with stigmasterol for 6 h, followed by incubation with 10 μg/mL Dil‐ox‐LDL (YB‐0010; Yiyuan Biotech) and stigmasterol for an additional 4 h. Fluorescence images were captured using a laser scanning confocal microscope (Zeiss LSM 880). Quantification of Dil‐ox‐LDL uptake was performed using a CytoFLEX LX flow cytometer (Beckman Coulter).

### Cholesterol efflux

Cells were loaded with NBD‐cholesterol (5 μg/mL) for 12 h, followed by incubation for 4 h in no phenol red culture medium (TBD) with 0.2% BSA and HDL (50 μg/mL). The control group was treated with a medium containing 0.2% BSA but no HDL. After incubation, the culture medium was collected, and cells were lysed with 0.3 M NaOH. The fluorescence intensity of both supernatant and cell lysates was measured by fluorescence microplate reader. Cholesterol efflux rate was calculated using the following formula: Efflux (%) = supernatant counts/(supernatant counts + cell lysate counts) * 100. Cholesterol efflux was also observed using a confocal microscope (Zeiss LSM 880).

### Immunofluorescence

Tissue sections or cell samples were fixed with 4% paraformaldehyde and permeabilized with 0.05% Triton X‐100 for 15 min. After blocking with blocking solution (Beyotime Biotechnology), samples were incubated with primary antibodies overnight at 4°C. The following day, slides were incubated with fluorescently labeled secondary antibodies for 1 h, followed by nuclear staining with DAPI. Fluorescent images were acquired using a confocal microscope (Zeiss LSM 880). Primary antibodies used in this study included anti‐CD68, anti‐CD163, anti‐α‐SMA, anti‐F4/80, and anti‐ACAT2. Neutral lipids were stained using BODIPY 493/503 (Thermo Fisher Scientific).

### Statistical analysis

Data were analyzed using ImageJ and GraphPad Prism 8 software. Results are presented as mean ± SD. Statistical comparisons among multiple groups were determined using one‐way ANOVA, followed by Tukey's multiple comparison test for multiple comparisons. Unpaired *t*‐test was used to detect significant differences between the two groups. Statistical significance was indicated as follows: *p < 0.05, **p < 0.01, ***p < 0.001, and ****p < 0.0001.

## AUTHOR CONTRIBUTIONS

**Baiyi Lu**: Supervision; methodology; writing–original draft; visualization; funding acquisition. **Fan Xiao**: Methodology; data analysis; writing–original draft; writing–review and editing. **Qinjun Zhang**, **Yao Xie**, **Mengmeng Wang**: Investigation; data analysis; conceptualization. **Jesus Simal‐Gandara**, **Yan Liu**, **Weisu Huang**, **Thomas Efferth**, **Jianfu Shen**, **Jianbo Xiao**: Validation. **Baiyi Lu**: Supervision; visualization; funding acquisition. All authors have read the final manuscript and approved it for publication.

## CONFLICT OF INTEREST STATEMENT

The authors declare no conflicts of interest.

## ETHICS STATEMENT

Animal experiments were conducted according to the instructions of Zhejiang Chinese Medical University (Approval No: IACUC‐20190624‐04, IACUC‐20220418‐15).

## Supporting information


**Figure S1**. Different effects of dietary PS/COPs/POPs on atherosclerosis plaque and lipid levels in serum. **Figure S2**. Body weight and inflammatory cytokines production in mice. **Figure S3**. Summary of sequencing results from the 10x genomics platform. **Figure S4**. Identification of aortic cells from *ApoE* ^−/−^ mice. **Figure S5**. Non‐immune cells composition. **Figure S6**. Gene expression of macrophage and SMC subpopulations. **Figure S7**. KEGG analysis of 7 macrophage subpopulations. **Figure S8**. KEGG analysis of 5 SMC subpopulations. **Figure S9**. Cytotoxicity of different concentrations of phytosterol to RAW264.7 cells. **Figure S10**. Foam cell formation in macrophages under different phytosterol concentrations. **Figure S11**. Cytotoxicity of A7r5 cells treated with different concentrations of stigmasterol. **Figure S12**. Effects of stigmasterol on body weight and food intake in *ApoE* ^−/−^ mice.

**Table S1**. Composition of phytosterols, cholesterol oxidation products and phytosterol oxidation products in diets. **Table S2**. Cell counts and percentage of macrophage subtypes. **Table S3**. Cell counts and percentage of smooth muscle cell subtypes.

## Data Availability

The data that support the findings of this study are openly available in the NCBI Sequence Read Archive (SRA), reference numbers PRJNA1298524 (https://www.ncbi.nlm.nih.gov/bioproject/?term=PRJNA1298524). The data for analysis are saved in GitHub (https://github.com/qinle77/Zju_Bylu/tree/master). Supplementary materials (tables, figures, graphical abstract, slides, videos, Chinese translated version and update materials) may be found in the online DOI or iMeta Science http://www.imeta.science/imetaomics/.
