# Supplementary material for: Stigmasterol attenuates atherosclerosis by inhibiting inflammatory signaling and foam cell formation
Source: IMetaOmics. 2025 Oct 1;2(4):e70056. doi: 10.1002/imo2.70056 (PMC12805992; doi:10.1002/imo2.70056)
Supplement: Supplementary file 1 — Figure S1. Different effects of dietary PS/COPs/POPs on atherosclerosis plaque and lipid levels in serum. Figure S2. Body weight and inflammatory cytokines production in mice. Figure S3. Summary of sequencing results from the 10x genomics platform. Figure S4. Identification of aortic cells from ApoE −/− mice. Figure S5. Non‐immune cells composition. Figure S6. Gene expression of macrophage and SMC subpopulations. Figure S7. KEGG analysis of 7 macrophage subpopulations. Figure S8. KEGG analysis of 5 SMC subpopulations. Figure S9. Cytotoxicity of different concentrations of phytosterol to RAW264.7 cells. Figure S10. Foam cell formation in macrophages under different phytosterol concentrations. Figure S11. Cytotoxicity of A7r5 cells treated with different concentrations of stigmasterol. Figure S12. Effects of stigmasterol on body weight and food intake in ApoE −/− mice. [file IMO2-2-e70056-s002.docx]

**Supporting information to:**

**Stigmasterol** **attenuates atherosclerosis by inhibiting inflammatory signaling and foam cell formation**

**Running title: Differentiation and lipid metabolism in foam cells**

Baiyi Lu^1,2^**^*^**, Fan Xiao^1,2,^, Qinjun Zhang^1,2,^, Yao Xie^3^, Mengmeng Wang^1,2^, Jesus Simal-Gandara^4^, Yan Liu^1,2^, Thomas Efferth^5^, Weisu Huang^6^, Jianfu Shen^1,2^, Jianbo Xiao^4^

^1^College of Biosystems Engineering and Food Science, National-Local Joint Engineering Laboratory of Intelligent Food Technology and Equipment, Key Laboratory for Agro-Products Nutritional Evaluation of Ministry of Agriculture and Rural Affairs, Key Laboratory of Agro-Products Postharvest Handling of Ministry of Agriculture and Rural Affairs, Zhejiang Key Laboratory for Agro-Food Processing, Zhejiang International Scientific and Technological Cooperation Base of Health Food Manufacturing and Quality Control, Zhejiang University, Hangzhou, 310058, China

^2^Ningbo Research Institute, Zhejiang University, Ningbo, 315100, China

^3^Department of Cardiology, The Second Affiliated Hospital, Zhejiang University School of Medicine, Cardiovascular key Lab of Zhejiang Province, Hangzhou, 310009, China

^4^Department of Analytical Chemistry and Food Science, Faculty of Food Science and Technology, University of Vigo-Ourense Campus, Ourense, 32004, Spain

^5^Department of Pharmaceutical Biology, Institute of Pharmacy and Biomedical Sciences, Johannes Gutenberg University, Staudinger Weg Mainz, 55128, Germany

^6^Zhejiang Economic & Trade Polytechnic, Department of Applied Technology, Hangzhou, 310018, China.

***Correspondence:** [bylu@zju.edu.cn](mailto:bylu@zju.edu.cn) (Baiyi Lu)

**Supplementary figures**


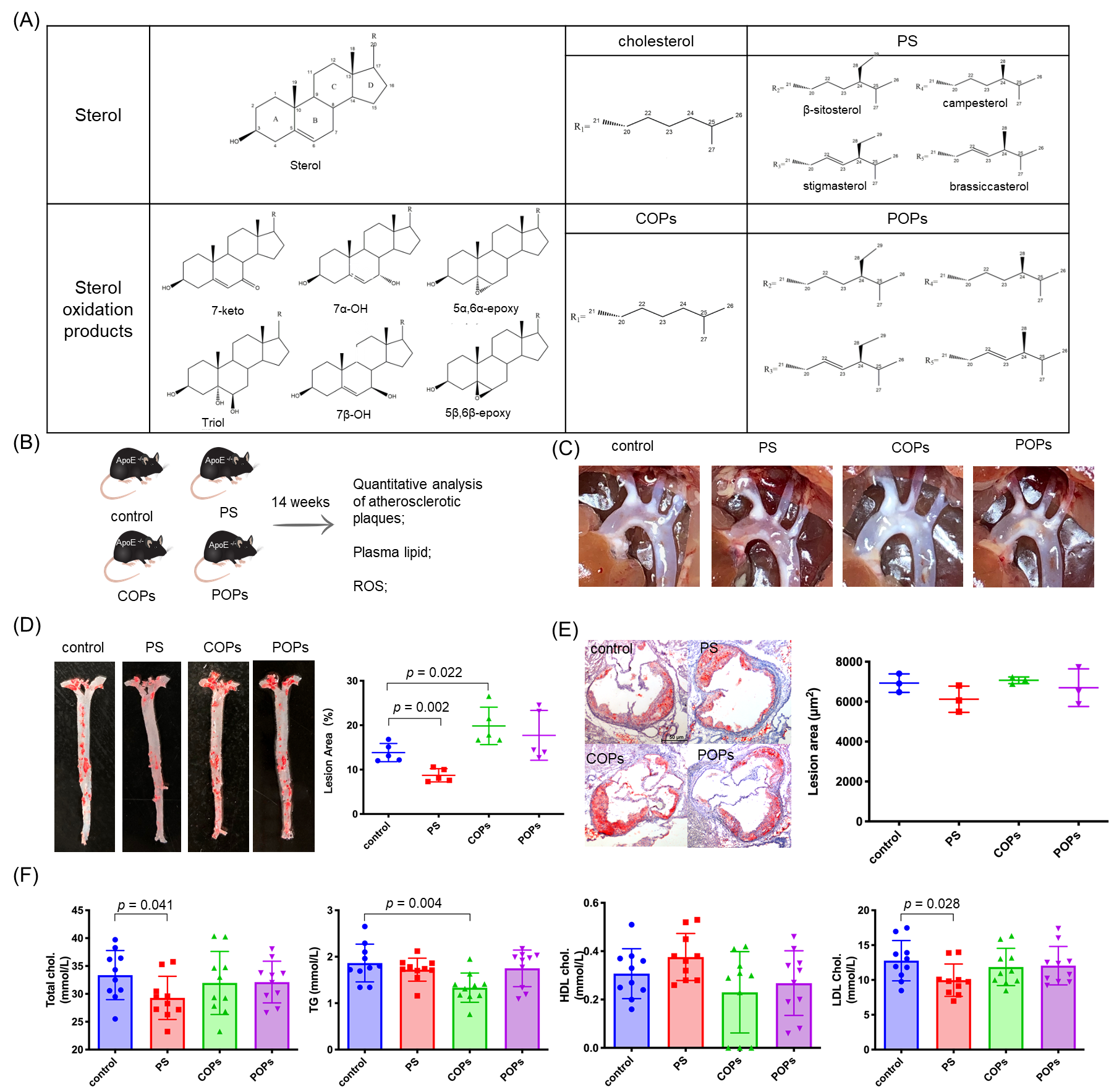


**Figure S1 Different effects of dietary PS/COPs/POPs on atherosclerosis plaque and lipid levels in serum.** (A) Structure of sterols and sterol oxidation products. (B) Schematic overview of experimental design. (C) Representative in situ images of atherosclerotic plaque in the aortic arch of *ApoE*^-/-^ mice. (D) Representative gross Oil red O staining of the aorta. *n* = 5. (E) Representative lipid and haematoxylin-stained aortic root sections (magnification 40×). *n* = 3. (F) Levels of total cholesterol, triglyceride, HDL cholesterol, and LDL cholesterol in plasma. *n* = 10. The data are presented as the mean ± SD. **p* < 0.05, ***p* < 0.01, ****p* < 0.001, *****p* < 0.0001.


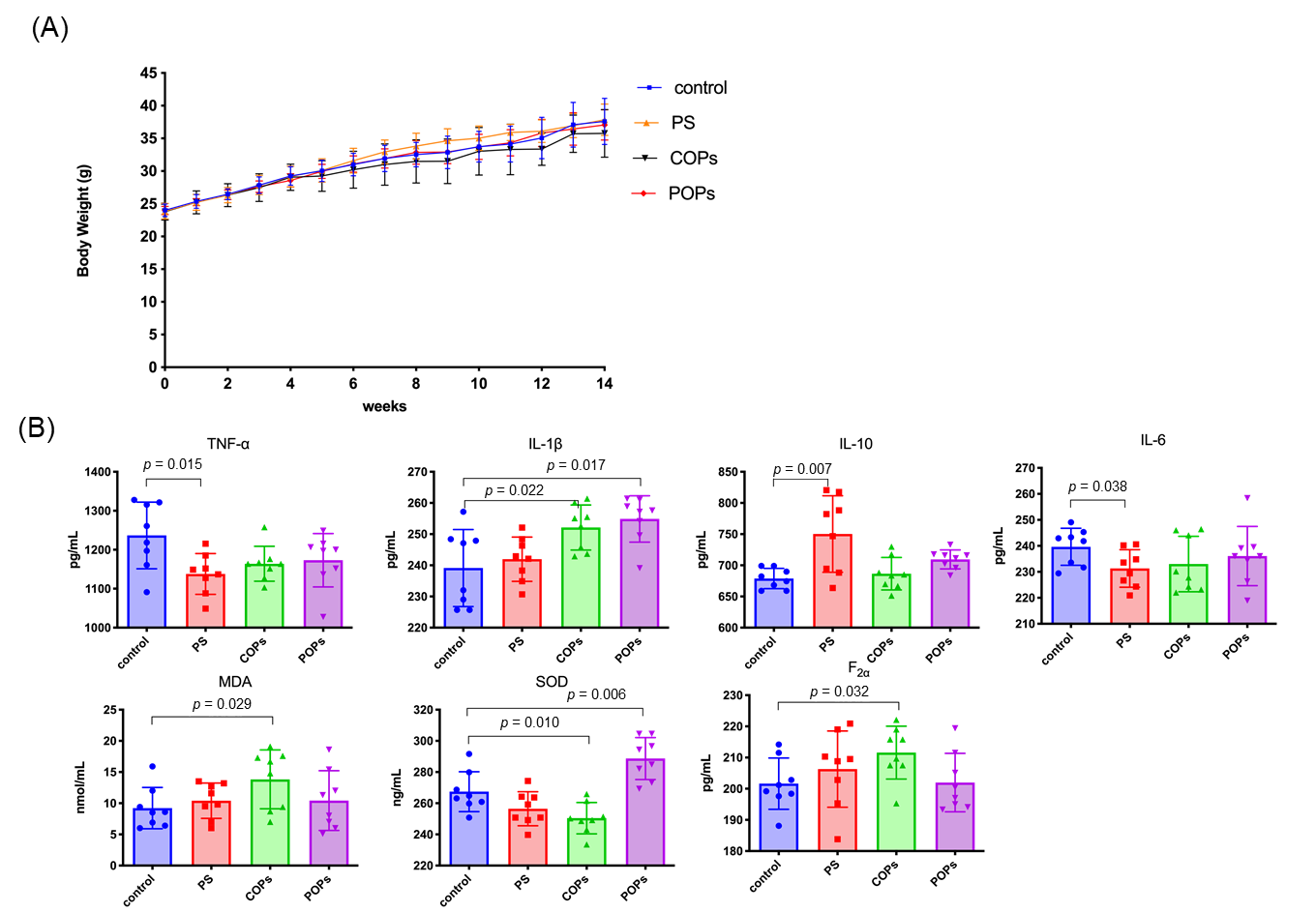
**Figure S2 Body weight and inflammatory cytokines production in mice.** (A) Body weight. (B) Inflammatory cytokines production in mice fed a control (*n* = 8), phytosterols (PS, *n* = 8), cholesterol oxidation products (COPs, *n* = 8), or phytosterol oxidation products (POPs, *n* = 8) diet at the 14th week with a western diet. The data are presented as the mean ± SD. Multi-group comparison was measured by one-way ANOVA. **p* < 0.05, ***p* < 0.01, ****p* < 0.001, *****p* < 0.0001.


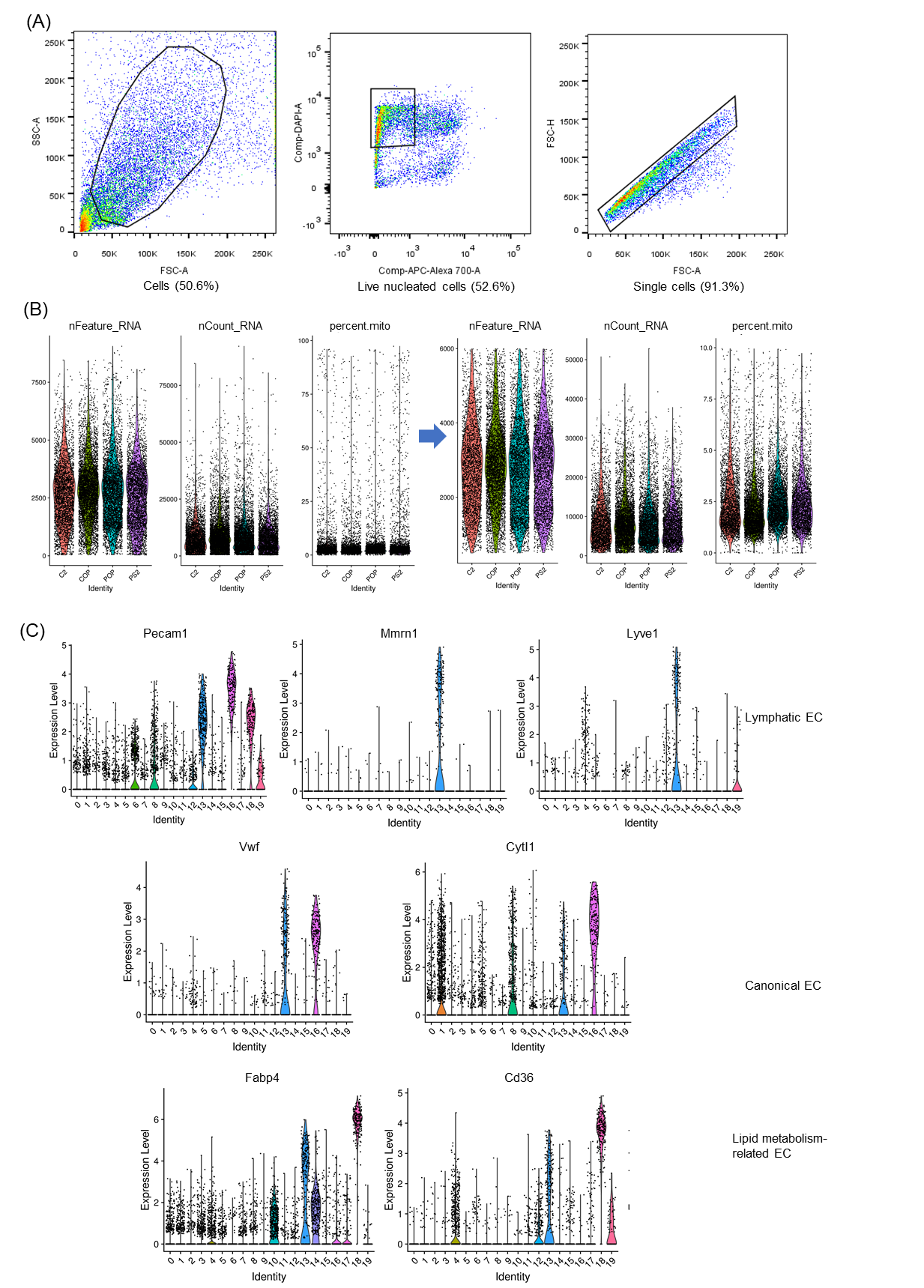


**Figure S3 Summary of sequencing results from the 10x genomics platform.** (A) Gating strategy to obtain live single cells by flow cytometry. (B) Estimated number of cells, reads, genes, and UMI. (C) Markers used to identify endothelial cell (EC) subpopulations.


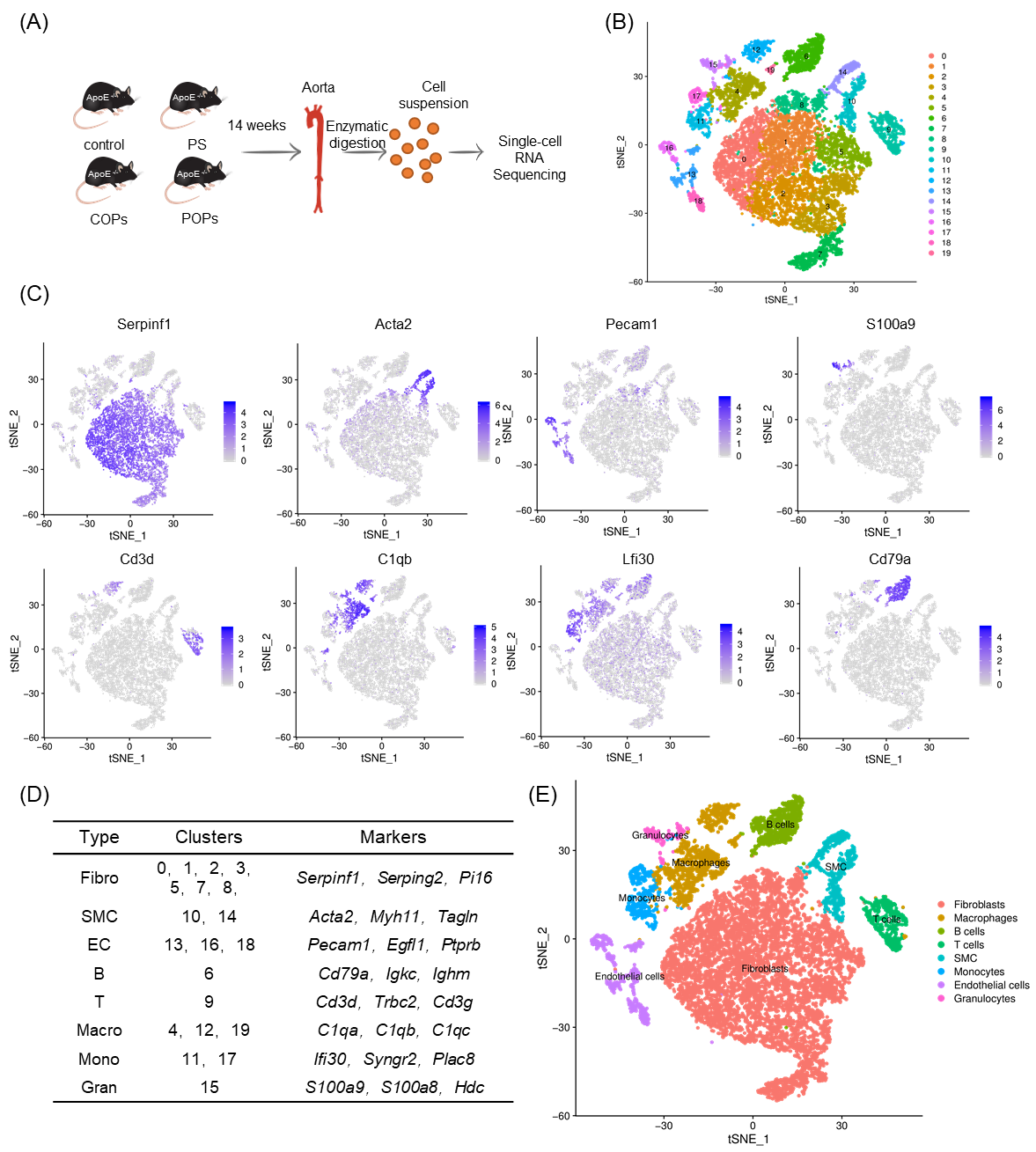


**Figure S4 Identification of aortic cells from *ApoE*^-/-^ mice.** (A) A schematic representation of how experiments are designed. (B) T-distributed stochastic neighbor embedding (t-SNE) visualization of clusters in aortic cells from 4 groups of *ApoE*^-/-^ mice. (C) Feature plot of markers of each cell type. (D) Cluster and major cell markers. (E) t-SNE plot of cell types in aortic cells from *ApoE*^-/-^ mice groups.


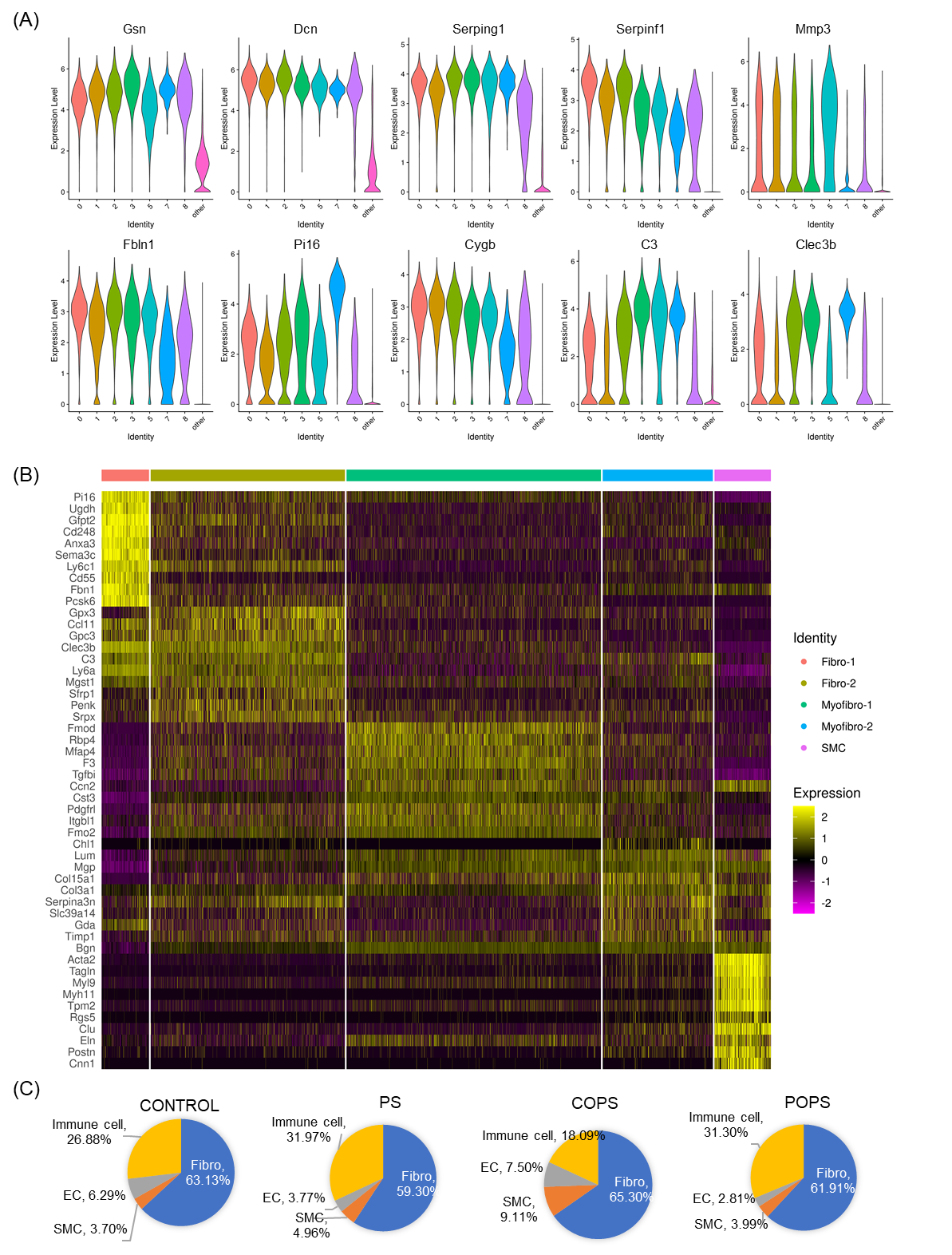


**Figure S5 Non-immune cells composition.** (A) Specific genes of fibroblasts. (B) Heatmap of TOP 10 genes with specific expression for each fibroblast subpopulations. (C) Fraction of fibroblasts (Fibro), immune cells, smooth muscle cells (SMC), and EC in each group.


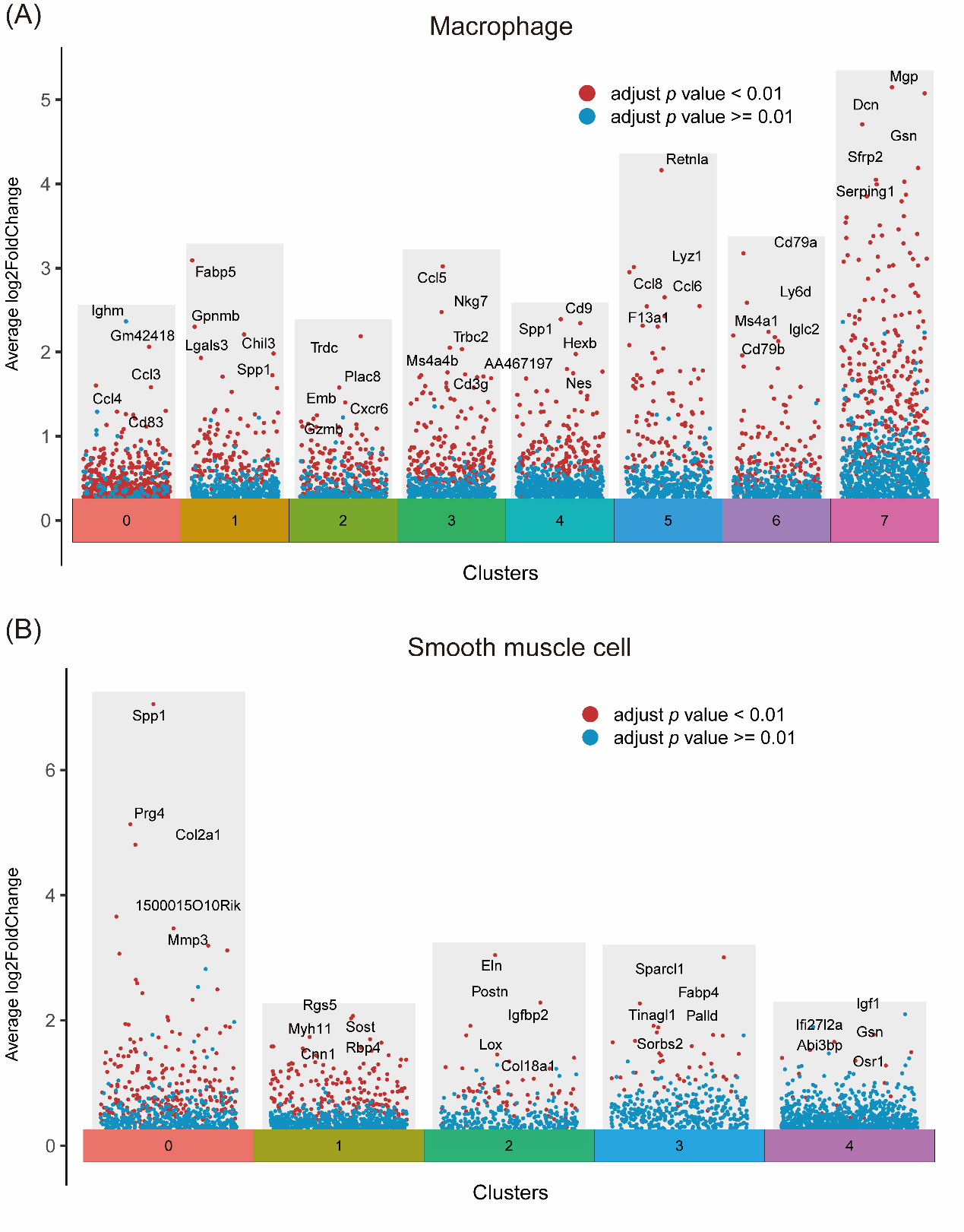


**Figure S6 Gene expression of macrophage and SMC subpopulations.** The vertical axis represents the log2-transformed fold change in gene expression between the target cell population and other cell populations' average gene expression. The horizontal axis represents the proportion of gene expression in the target cell population minus the proportion of gene expression in other cell populations. (A) Specific genes of macrophage subgroups. (B) Specific genes of SMC subgroups.


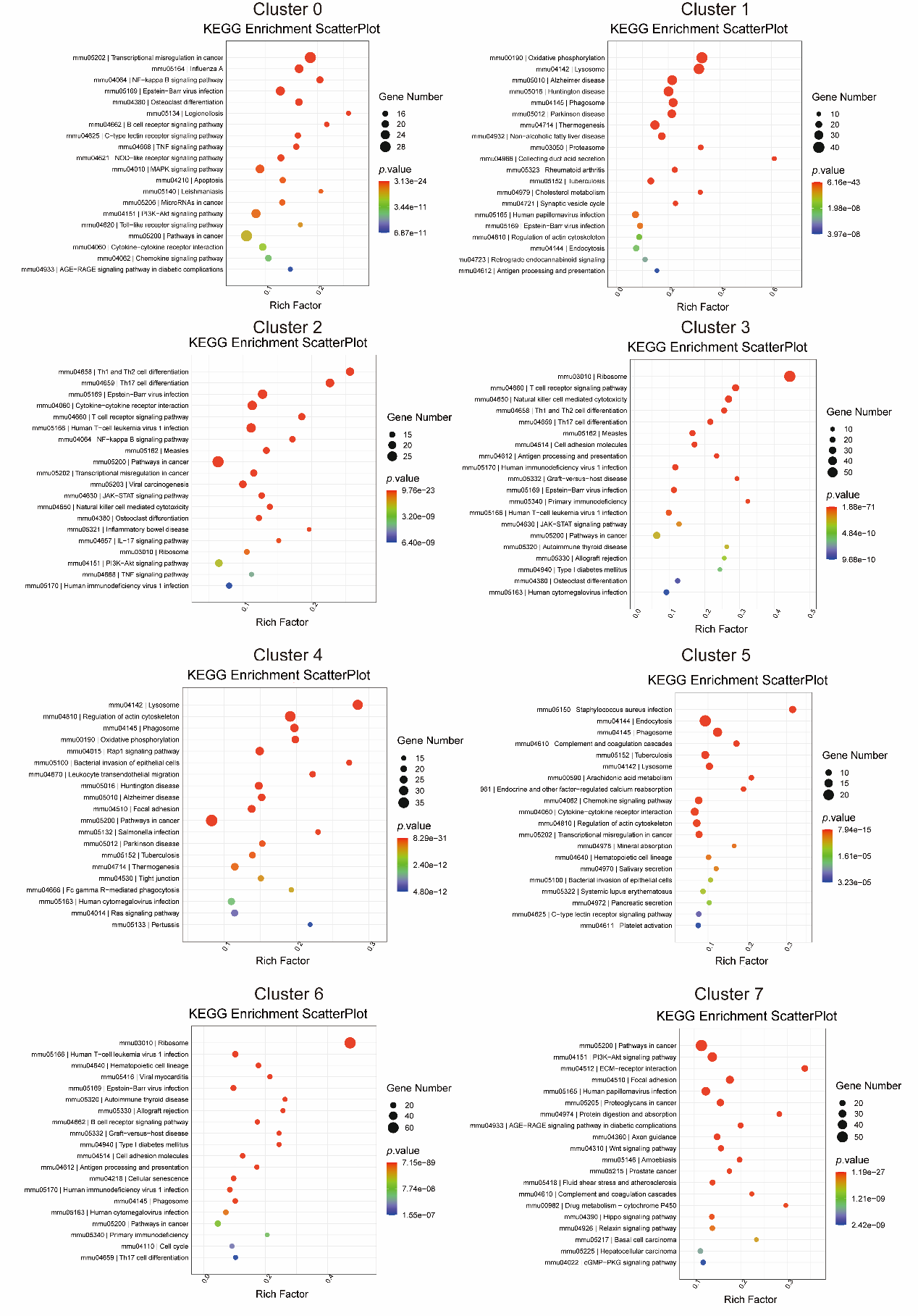


**Figure S7 KEGG analysis of 7 macrophage subpopulations.** Cluster 0: Inflammatory Macro, Cluster 1, 4: Trem2^+^ Macro, Cluster 2: Trdc^+^ Macro, Cluster 3: Cd3g^+^ Macro, Cluster 5: Res-like Macro, Cluster 6: Ebf1^+^Cd79a^+^ Macro, Cluster 7: Dcn^+^ Macro.


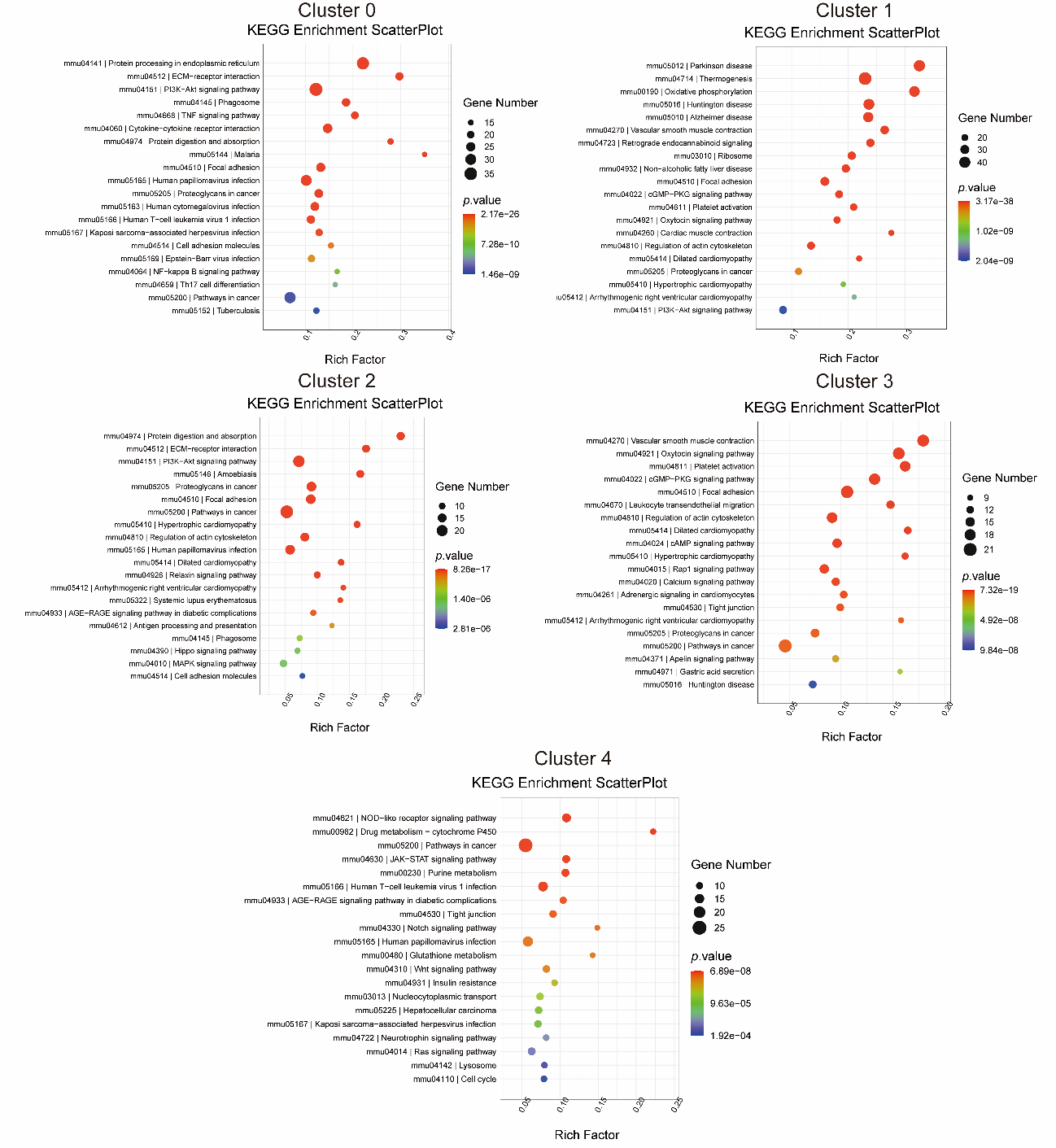


**Figure S8 KEGG analysis of 5 SMC subpopulations.** Cluster 0: SEM, Cluster 1: SMC, Cluster 2: FC-like SMC, Cluster 3: SMC, Cluster 4: Lgf1^+^ SMC.


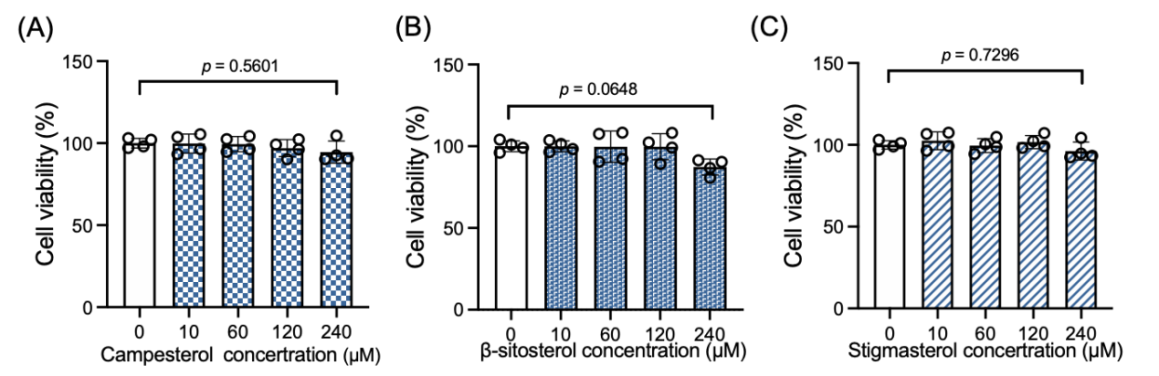


**Figure S9 Cytotoxicity of different concentrations of phytosterol to RAW264.7 cells.** (A) Cell viability of Campesterol. *n* = 4. (B) Cell viability of β-sitosterol. *n* = 4. (C). Cell viability of Stigmasterol. *n* = 4. The data are presented as the mean ± SD. Multi-group comparison was measured by one-way ANOVA.

**
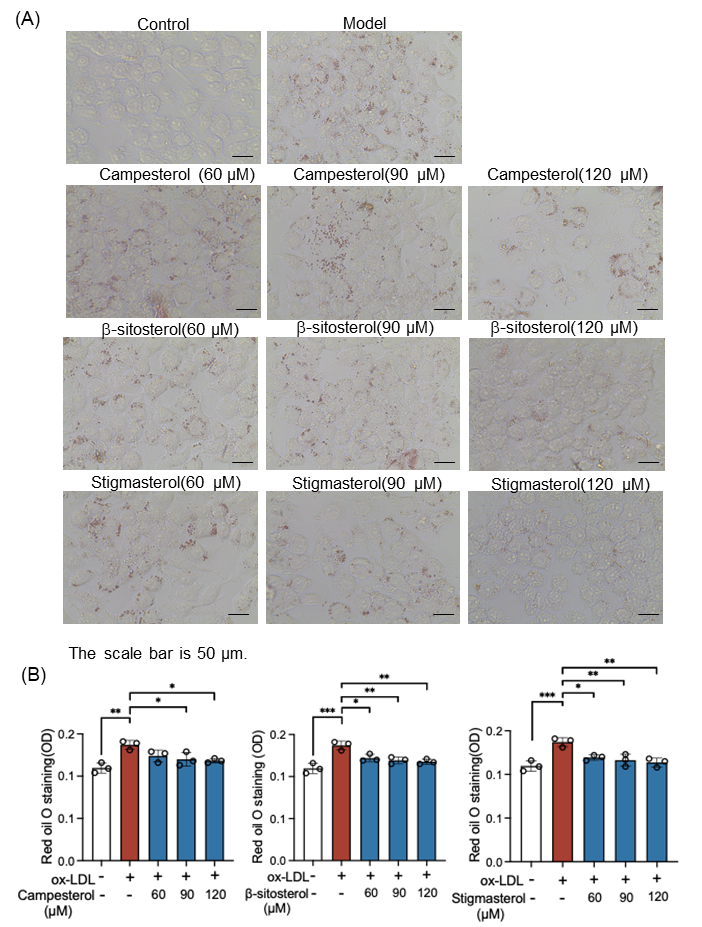
Figure S10** **Foam cell formation in macrophages under different phytosterols concentrations.** Cells were incubated with phytosterol (60 μM, 90 μM, and 120 μM) for 6 h, then co-incubated with ox-LDL for 24 h. (A) Morphologic change of foam cells stained by Oil Red O (ORO). (magnification 200×). The scale bar in the figure is 50 μm. (B) Quantitation of the extracted ORO dyes from the stained macrophages that were treated with ox-LDL. *n* = 3. The data are presented as the mean ± SD. Multi-group comparison was measured by one-way ANOVA. **p* < 0.05, ***p* < 0.01, ****p* < 0.001.


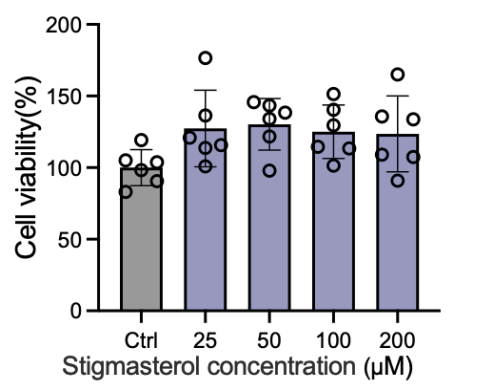


**Figure S11** **Cytotoxicity of A7r5 cells treated with different concentrations of stigmasterol.** Cells were cultured in the medium with the relative concentration of stigmasterol for 24 h. Cell cytotoxicity was evaluated by the CCK8 kit. *n* = 6. The data are presented as the mean ± SD. Multi-group comparison was measured by one-way ANOVA.


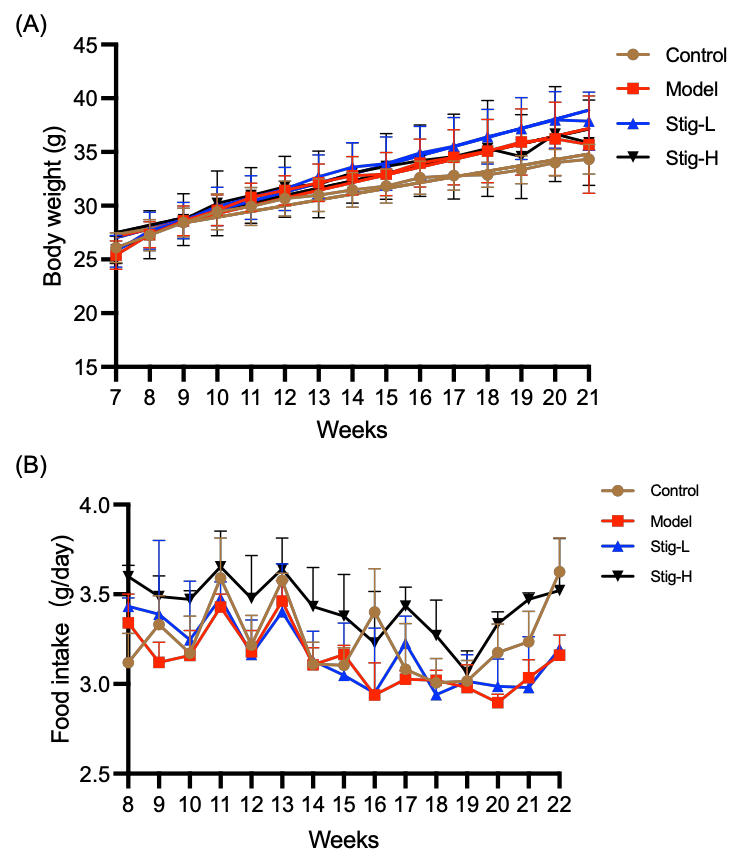


**Figure S12 Effects of stigmasterol on body weight and food intake in *ApoE^-/-^* mice.** Mice fed a control (*n* = 15), model (*n* = 15), stigmasterol low dose (*n* = 15), and stigmasterol high dose (*n* = 15) diet from 7 weeks to 21 weeks old. (A) Body weight. (B) Food intake.
